# Experiences of family hope and hopelessness in the context of pregnancy and care for high-risk newborns^*^

**DOI:** 10.1590/1518-8345.7906.4834

**Published:** 2026-07-24

**Authors:** Bruna Camargos de Lima Ramos, Renata de Oliveira Costa, Elysângela Dittz Duarte, Zaida Borges Charepe, Patrícia Pinto Braga

**Affiliations:** 1 Universidade Federal de São João del-Rei, Curso de Enfermagem, Divinópolis, MG, Brazil.; 2 Universidade Federal de Minas Gerais, Departamento de Enfermagem, Belo Horizonte, MG, Brazil.; 3 Universidade Católica Portuguesa, Escola de Enfermagem, Lisboa, Lisboa, Portugal.

**Keywords:** Hope, Family, High-Risk Pregnancy, Infant, Newborn, Nursing Research, Family Nursing.

## Abstract

**Objective:**

to understand family experiences of hope, from pregnancy to the care for high-risk newborns.

**Method:**

qualitative research, guided by the Theoretical Model for Understanding the Complex Nature of Hope, which took place in a multiprofessional outpatient clinic for high-risk newborn care. A total of 28 individuals from 14 families participated through oral history interviews. This allowed for the construction of family narratives, which were submitted to deductive thematic analysis.

**Results:**

family experiences of hope are established based on four dimensions: understanding, judgment, and interpretation about hope; affective aspects and feelings regarding the lived experience; actions, behaviors, and strategies that strengthen family hope; and influence of context and life situations. These are influenced by the temporality in which the family finds itself. At the beginning of the experience of risk, hope appears fragile and uncertain, with negative feelings and desires for better days. Over time, strategies, such as spirituality and intrapersonal relationship skills, are strengthened and maintained, reframing feelings.

**Conclusion:**

understanding family experiences of hope and hopelessness can enable healthcare providers to strengthen and contribute to a more positive experience in the presence of gestational and neonatal risk.

## Highlights

**(2)** Hope is an essential resource in the context of gestational and neonatal risk. **(2)** Family hope is dynamic and influences temporality. **(3)** The experience of family hope is influenced by relationships, beliefs, and emotions. **(4)** Nursing should assist families in strengthening hope

## Introduction

In the context of high-risk pregnancy and neonatal care, family members face complex situations, such as the confirmation of gestational risk, maternal or neonatal hospitalization, fears related to the prognosis, and frequent demands for healthcare^1^ ^-^ ^2^. The impact of this experience on families is multifaceted, affecting physical, psychological, and social dimensions. High-risk pregnancies often culminate in premature births, low birth weight, and clinical complications in cases of congenital anomalies, which require intensive neonatal care and can result in long-term health problems for the newborn^3^.

Gestational and neonatal risk affects the family system, and it is pertinent to note that family-centered care (FCC) has been associated with improvements in clinical outcomes and can contribute to fostering a culture that values partnerships with families^4^ ^-^ ^5^. From this perspective, valuing FCC becomes a pertinent strategy for addressing hope for this group.

Among family members, mothers in particular report greater emotional burden due to long-term care needs^4^, presenting higher rates of anxiety, post-traumatic stress disorder, and depressive symptoms^3^ ^-^ ^4^.

Although studies exist on the physical and emotional impacts of high-risk pregnancies and neonatal care, it is necessary to understand how hope is experienced, constructed, and reframed by families over time. 

Hope, according to the Theoretical Model for Understanding the Complex Nature of Hope^6^, is characterized as an experience of meaning and purpose in life, involving expectations of improvement, desires for change, and positive thoughts. This model provides a theoretical framework for analyzing the dimensions of hope, based on 5 dimensions, including temporality, cognition, affect, behavior, and context^4^ ^-^ ^6^. In summary, the main ideas of these dimensions are: a) temporal, which involves the experience of each individual in the present, past, and future time, in which hope is directed toward the future, although influenced by the past and present; b) cognitive, related to the process by which the person desires, imagines, interprets, and judges regarding hope, has the perception of the realistic desire for the future; c) affective, which encompasses a range of emotions and feelings, sometimes opposite, among which there is an attraction to the goal to be achieved; d) behavioral, which relates to the action that seeks the goal pertaining to hope in the physical, psychological, social, and spiritual realms; and e) contextual, which refers to the life situations and environment that surround, influence, and constitute part of the person’s hope^6^.

Similar to other studies that address it^7^ ^-^ ^10^, the model provided a basis for categorizing and interpreting the experiences of the participating families in this study, facilitating the identification of patterns and recurring themes under the mentioned dimensions.

Understanding family experiences of hope can enable healthcare providers and nurses to strengthen and contribute to a more positive experience in the face of gestational and neonatal risk^3^. Strategies such as active listening, emotional support, respect for religious and cultural beliefs, and encouragement of participation in care indicate the promotion of family hope. Recognizing that the family experience, hope, and hopelessness change over time is essential to offer more constructive and centered care to this group^3^ ^,^ ^6^.

Given the above, the following guiding question arose: how does the family experience of hope present itself, from pregnancy to the care of the high-risk newborn? This study aimed to understand family experiences of hope, from pregnancy to the care of the high-risk newborn.

## Method

### Study design

This interpretive qualitative study was guided by the Theoretical Model for Understanding the Complex Nature of Hope^6^. The model provided a conceptual framework of the dimensions that underpin hope (temporality, cognition, affect, behavior, and context), supporting the theoretical and methodological path and data analysis.

### Study participants and setting

The participants were 28 family members (including mother, father, grandmother, aunt, and sister) from 14 families of high-risk newborns, whose mothers had a pregnancy assessed as high-risk^11^. As the study aimed to understand family experiences, in addition to the primary caregiver, other family members indicated by the primary caregiver were interviewed. Interviews with participants were guided by the same script and at different times, individually and privately. The study settings were the homes of these families and the multidisciplinary outpatient clinic of the Advanced Early Intervention Program (PIPA), in Divinópolis-MG, Brazil, for the care of newborns at risk. The service follows high-risk newborns and children until they complete 2 years of age and performs a mean of 192 appointments per trimester.

The inclusion criteria for participation were family members of children between 6 and 12 months of age during data collection, with a history of neonatal risk, allowing sufficient time for the experience to be shared through oral histories and establishing a time limit between birth and the interview. Families were excluded if the mothers were under 18 years of age, classified as high-risk during pregnancy due to issues such as depression or psychiatric disorders, or had depression or mental distress up to 45 days after childbirth. These criteria are justified because the interview could mobilize memories, distress, or embarrassment for the women and/or family members.

From the analysis of 146 medical records from the outpatient clinic, 60 families were pre-eligible. First, an approach was made through a preliminary telephone contact, explaining the research objective, and of these, 24 families did not respond to the invitation and five refused.

### Data collection

The initial contact was made by telephone with the primary caregiver, who in this research were mothers (13) and a father (1). Interviews were scheduled at PIPA or at home, respecting the participant’s desire and availability. At the end of the interview, the participant was asked to indicate other family members who also cared for the child from the moment the neonatal risk was discovered until the time of the interview. At this point, the concept of family adopted in the study was clarified, so that the participant could identify people involved in the child’s care. This process allowed for the identification of other family members to participate later in the interviews. In this research, family is understood as a relational structure composed of individuals who have affective and/or biological bonds with each other, with mutual commitment, identity of life projects, and common purposes^12^.

For data collection, a thematic oral history interview^13^ was conducted, supported by a semi-structured script with questions regarding the characterization of family composition, gestational and neonatal risk, and the internal relationship of the family context. The script also included open questions related to family hope experiences, considering the different dimensions of the theoretical model, during the period of pregnancy and care for the high-risk newborn, and related to the family interrelational pattern. Fieldwork was conducted between December 2021 and March 2022 by the first author, who is a nurse and has experience in qualitative research and fieldwork, and was assisted by an undergraduate nursing student from the proposing institution. Neither had any relationship with the potential participants or research settings.

The mean duration of each thematic oral history interview was approximately forty-five minutes. At the time of the interview, only the interviewers and the participant were present.

The interruption of interviews and the definition of the participant limit occurred based on theoretical data saturation^14^. For this purpose, content analysis of the interviews was adopted concomitantly with data collection^14^ ^-^ ^15^. Theoretical saturation of the data was determined at the 14^th^ family, when 28 interviews had already been conducted.

The challenges involved in the sampling process by saturation were recognized; therefore, during the analysis work, concomitant with data collection, the following strategies were adopted to minimize inaccuracies: (a) alignment and in-depth study of the framework adopted by the researchers before its applicability; (b) validation of the code dictionary developed from the framework; (c) coding with intercoder validation and meetings for analysis and consensus; (d) attentive look at each narrative, identifying the similarities and singularities of the lived experiences; (e) identification of the representativeness of the adopted framework based on the narratives; (f) capacity to respond to the study objective.

### Data treatment and analysis

After the interviews were transcribed, narratives were constructed for each family, which were submitted to deductive thematic analysis^13^ ^,^ ^16^. For this purpose, a code dictionary was developed, encompassing the theoretical model^17^. To validate the code dictionary, one narrative was analyzed simultaneously by three researchers with expertise in the framework. Four rounds of coding were necessary for adjustments and theoretical alignments in the code dictionary. Figure 1 exemplifies the coding process of some narratives, supported by the model’s dimensions.

**Figure 1:** Examples of narrative coding based on the dictionary

**Code**	**Narrative statements**
**Cognition**: the individual’s appraisal, related to the lived process, through which they interpret, judge, desire, and imagine hope; presence or absence of realistic desires regarding the future.	*Hope is what keeps us alive. I think that is it. I am almost certain that if we do not have hope, there is no point in living. So, based on that question, I think I always had the hope that everything would work out* **(Grandmother 9).** *Hope is to believe and to transform. Better days* **(Maternal Sister 3).**
**Behavioral**: actions directed toward hope in the physical, psychological, social, and spiritual realms. Strategies that can promote, strengthen, or weaken family hope.	*To have an expectation of hope when we discovered the risk, my mother-in-law and I went to the chapel and knelt, prayed. The strategy to have hope was to believe in God* **(Mother 2).** *The strategy used to have hope, the most certain one for us was unity, being together, not leaving the person alone. Sometimes I would leave there, their mother was crying, I would talk to her: It will be okay! I think the strategy we used was that, it was unity, grit with each other there in faith, there so that everything would work out* **(Father 13).**

The process of coding a first narrative then followed, which was conducted by two researchers independently and subsequently presented to a third researcher. Four rounds of coding were necessary to identify congruence in the process. From then on, the narratives were coded by the researchers, and situations of divergence or doubt were resolved in meetings. This process ensured the reliability of the coding^16^. After the coding process, it was possible to perform groupings of codes to compose the categories, in order to portray and present the content of the deductive analysis of the narratives.

Regarding the criteria of scientific rigor, credibility was ensured through rigorous analyses conducted simultaneously with the data collection process, supported by the narratives and by the construction of the instruments related to the hope relationship. Reliability was present through the detailed description of the method, following the Consolidated Criteria for Reporting Qualitative Research (COREQ) checklist. Confirmability was contemplated and guaranteed, considering the presentation of the study’s strengths and limitations^18^ ^-^ ^20^.

### Ethical aspects

The study was conducted according to the ethical aspects that guide the development of research with human subjects in the country, having been approved by the Research Ethics Committee of the proposing institution under opinion number 5,119,278 and Certificate of Presentation for Ethical Appreciation (CAAE) number 51866821.4.0000.5545. All participants were informed about the research objectives and completed the consent process by signing the consent form before the start of data collection. To guarantee anonymity, each participant’s name was replaced by their relationship to the child, followed by the interview order, for example: mother 1, maternal grandfather 1, and so on.

## Results

The research data analysis allowed for a synthesis of the presentation of the family members. Of the 28 study participants, 46.4% (13) were biological mothers, 21.4% (6) fathers, 14.3% (4) aunts, 10.7% (3) grandmothers, 3.6% (1) godmother, and 3.6% (1) sister. The age range of the participants varied between 25 and 56 years.

Among the risk conditions presented by the newborns, the following were identified: 15.7% (3) premature, 47.3% (9) prematurity associated with twinning, 10.5% (2) congenital toxoplasmosis and 5.3% (1) for each of the following risks: rubella, pericardial effusion, neonatal sepsis, prematurity associated with congenital syphilis, prematurity associated with neonatal hydronephrosis, and ascites. The age of the children at the time of the research varied between 6 months and 1 year.

The gestational history of the mothers was accompanied by neonatal risk, and among the gestational risks, the following were identified: 14.3% (2) gestational hypertension associated with gestational diabetes; 14.3% (2) preeclampsia associated with twinning and 5.3% (1) for each of the following risks: gestational hypertension, gestational syphilis, toxoplasmosis, toxoplasmosis associated with gestational hypertension, hypothyroidism, preeclampsia associated with twinning and placental abruption, gestational diabetes associated with polyhydramnios, rubella, gestational hypertension associated with cardiac alterations and gestational diabetes associated with twinning.

Figure 2 presents the participants by family and their relationship to the newborn and the neonatal and gestational risks experienced by each family.

**Figure 2:** Family participants, their relationship to the newborn, and the gestational and neonatal risks. Divinópolis, MG, Brazil, 2025

Family	Participants	Neonatal Risk	Gestational Risk
F1	Father, Mother	Prematurity	HELLP syndrome* and diabetes
F2	Mother, Grandmother, Godmother	Congenital syphilis; prematurity	Syphilis
F3	Mother, Aunt	Congenital toxoplasmosis	Toxoplasmosis
F4	Mother, Sister	Congenital toxoplasmosis	Toxoplasmosis and hypertension
F5	Mother, Aunt	Prematurity	Preeclampsia and twinning
F6	Father	Prematurity, triplets	Hypertension, triplets, and diabetes
F7	Mother, Aunt	Prematurity	Hypertension and diabetes
F8	Mother, Father	Prematurity	Hypothyroidism
F9	Mother, Grandmother	Prematurity; twinning	Preeclampsia, twinning, placental abruption, COVID-19
F10	Mother, Father	Prematurity	Diabetes, polyhydramnios
F11	Mother, Aunt	Pericardial effusion	Hypertension and cardiac alterations
F12	Mother, Father	Congenital rubella	Rubella
F13	Mother, Father	Prematurity	Gestational diabetes and twinning
F14	Mother, Grandfather	Neonatal sepsis	Hypertension

*HELLP = Hemolysis, Elevated Liver enzymes, Low Platelets

The results of the deductive analysis will be presented based on the categories: Understanding, judgment, and interpretation about hope; Affective aspects and feelings regarding the lived experience; Actions, behaviors, and strategies that strengthen family hope; Influence of the life context on the family experience of hope and hopelessness.

### Understanding, judgment, and interpretation of hope

In general, the family members have different understandings of the word “Hope”. All participants reported that hope was present throughout the entire trajectory of caring for the child, from the discovery of the high-risk pregnancy until the present day. The understanding of hope changed and/or was strengthened based on the lived experience. From the discovery of the gestational and neonatal risk, as well as after the birth of the newborn, it was evidenced as a process of belief in a better future, in the desire and wait for better days.

*When I discovered the risk, I had hope that everything would change. That better days could come*

(Mother 10)

*When we realize that the baby was born with the risk, then we understand what hope is. Always thinking of better days*

(Grandmother 9)

*I had a lot of hope there was first for the delivery to go well, you know, for the mother, I already knew that they were premature, you know, that something could happen to them. So I think that is it, it is hope for everything to work out, for them to be born well, born healthy, even though we went through all that in the ICU, I always had this hope that everything would work out*

(Father 13)

The analysis indicates that, over time, hope is interpreted as a positive experience that may have been strengthened by past experiences, for example, of discovering the gestational risk, recalling the idea of perseverance and an opportunity to believe in life again.

*Hope is everything that I lived through. Since I discovered my pregnancy, that they could be premature. And now I realize how important hope was for me.*

(Mother 13)

*Hope is what I believed in. It is trying to find the light deep inside, believing that everything would change. And, look, it did change .*

(Paternal Sister 4)

*It is a new opportunity to be living, every single day. They (twin daughters) gave me the opportunity to believe in life again. To fight for my family.*

(Father 6)

### Affective aspects and feelings in relation to the lived experience

The family experience in the trajectory of gestational and neonatal risks was constructed from ambiguous and contradictory feelings and emotions, favoring or not the maintenance of hope, and which changed over time. The analysis shows that there was a reframing of the lived situation, based on temporality, and this caused the family members to portray a change in their feelings.

In the context of the discovery of the risk involving the pregnancy and the newborn, feelings were initially reported as negative, with the presence of fear, anxiety, apprehension, despair, sadness, surprise, distrust, guilt, and anger.

*When I discovered her risk, I was very upset and very apprehensive*

(Mother 1)

*I think anxiety took me over. I became very anxious. This caused my hope to be shaken*

(Mother 8)

*I cannot speak of anything other than fear. Even when she was born, I was afraid; everything was strange*

(Father 12)

*Wow, I felt very guilty. To know that there would be, could be, a risk because of me*

(Mother 4)

Despite the uncertainties, after the birth of the newborn, feelings such as happiness, love, confidence, and relief were reported. For most family members, these feelings persist to the present day.

*Look, after the storm passed, when I saw that she was born well, relief, happiness, overtook me*

(Mother 14)

*What dominates me most to this day is the feeling of happiness, you know, for sure. When I saw that he was well after leaving the Intensive Care Unit (ICU), there were many tears of joy. Today, that’s it, only joy.*

(Maternal Grandfather 14)

Some family members report a maintenance of positive feelings, such as happiness and love, throughout the entire experience of caring for the newborn, from the discovery of the gestational and/or neonatal risk.

*When I discovered it, it was only love. I felt love the entire time. Until today.*

(Mother 10)

*I think that despite everything, the feeling that prevails the most is happiness. That made me have hope.*

(Father 10)

### Actions, behaviors, and strategies that strengthen family hope

The search for strategies and sources that strengthen hope, from pregnancy and after the birth of the newborn, involved mainly the practices of spirituality and skills for developing intrapersonal relationships. These strategies were present from the discovery of the risk and, over time, were strengthened.

The family members use strategies aimed at strengthening the spiritual and religious realm, through faith in God, by means of prayer and church attendance. These strategies were present from the discovery of the high-risk pregnancy and have been strengthened to the present day.

*What I used for hope was Jesus. I prayed a lot. It was God leading the way.*

(Mother 1)

*I used God to fight that, and then hope would come. And to this day, faith in God prevails, for sure.*

(Father 1)

*I always had a lot of faith, a lot of faith, you know? Since I discovered, discovered the risk. I prayed a lot, went to mass always, and today even more, I am grateful.*

(Mother 4)

*Strategies that helped me to have hope were to strengthen my spirituality, to seek out the church more, which helps me to this day (Mother 11).*

Intrapersonal relationships of involvement with oneself were also considered developed skills for having hope. The family members report having been their own source of support and action, mainly the mother, in the experience of pregnancy and birth of the high-risk newborn.

*I had to overcome; I was very strong there at that moment to be able to overcome.*

(Mother 6)

*We have to keep going with the pregnancy as far as possible, and everything will work out. That was when I was like, you know, having more hope.*

(Mother 11)

Resilience and emotional regulation, as well as self-confidence to continue caring for the neonate, were identified as essential for achieving the objective of maintaining hope in the present.

*It is positive thinking… it is, trying to really control my thoughts, you know? That helps me a lot.*

(Mother 4)

*After we went through everything, I say that I learned to be resilient in everything in life. At least all of this served as a lesson for my personal life too.*

(Maternal Aunt 5)

*After what I went through, after all the stress, after crying so much, I think I could get through anything now. I became stronger, more resilient .*

(Father 10)

### Influence of context and life situations on the family experience of hope and hopelessness

The hope experience was influenced by the context lived by the family members. The analysis allowed for the identification that the newborn’s hospitalization, associated with the COVID-19 pandemic, marked the hope experiences. The newborn’s hospitalization, due to the risk, was described as a period that threatened hope and even generated hopelessness. At this time, the families report that contact with the newborn was postponed and that it was difficult to return home without the presence of their child.

*They (twin daughters) were hospitalized, you know, that alone was bad. It is very difficult for us to have to leave there and leave them. Sometimes I left there disheartened… .*

(Father 13)

*The hospitalization caused my hope to be threatened. Seeing them (twin sons) hospitalized without knowing when they would be discharged, and if they would be discharged... I just cried and nothing more.*

(Mother 9)

The pandemic context negatively influenced the family experiences, restricting and postponing contact with the newborn after birth, increasing the feeling of insecurity and worry.

*Just this pandemic is bad, you know. We cannot do things properly… I could not see my son much or hold him, because everything was very fragile. I felt powerless.*

(Father 1)

*We got there, as if the worry about their case was not enough, there was COVID to make us feel*

(Mother 12)

*hopeless, right? A hospital environment is not good to begin with, with the pandemic then...*

(Father 10)

The analysis of the narratives showed that the experiences during pregnancy, birth, after delivery, and in neonatal care, including the present days with the child, contributed to the families reframing and constructing their “hope experience”, despite having experienced moments of hopelessness. In this way, the results present these experiences and their modifications over time.

**Figure 3:**
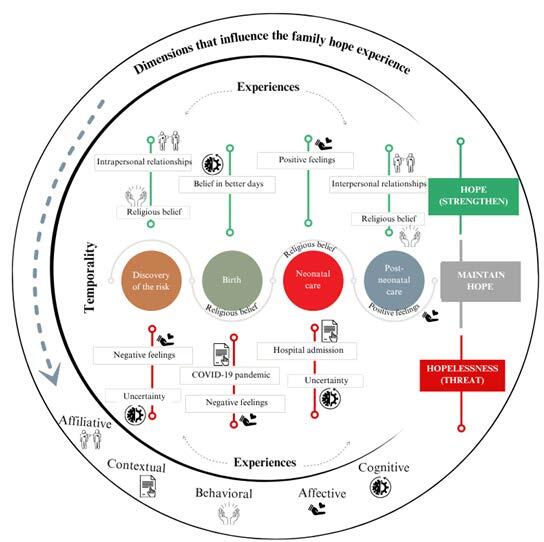
Family hope and hopelessness throughout high-risk pregnancy and neonatal care. Divinópolis, MG, Brazil, 2025

Figure 3 presents a synthesis of the main results of this investigation and highlights the elements that maintained, strengthened, or threatened hope in a path marked by changes and fluctuations related to hope and hopelessness.

## Discussion

The results showed that family experiences of hope are established based on the understanding of belief in a better future, ambiguous emotions, behaviors that seek to strengthen hope, and the life context, these being factors that change over time.

The analysis of the narratives allows us to state that the families’ stories present some similarities, such as the type of gestational or neonatal risk and the family structure. The hope experience of each family member was singular, influenced by the environment in which they are inserted, reflecting the contextual dimension set out in the adopted model^6^.

In this research, it became evident that maternal gestational risks were accompanied by neonatal risks. This reiterates the relevance of identifying the obstetric profile and maternal gestational risk of all newborns, favoring the prognosis, reducing the chances of morbimortality, hospital admission, and impairment of their health throughout life^21^ ^-^ ^22^.

The prevalent gestational risk (hypertension) and neonatal risk (prematurity) in this investigation are similar to those in other national and international realities^23^ ^-^ ^24^. This reaffirms the importance of a careful look at the factors that influence and predispose the mother-child group to negative outcomes.

The family members’ reports showed that the participants’ belief and desire for better days and the positive expectations regarding the future were strengthened and maintained over time, based on the entire lived context. From this perspective, it can be inferred that cognition^6^, expressed in understanding and judgments directed toward the future, favored a realistic construction, despite the maternal-child future being marked by uncertainties.

The moment of the newborn’s stabilization, after the family went through moments such as hospitalization, which threatened hope, helps to intensify the expectation that the risk will be overcome^25^. Therefore, despite the experience of risk, hope was maintained, strengthening a positive belief. The data seem to indicate that moments of crisis, such as the discovery of risk or neonatal hospitalization, function, depending on the interactions and support established, as devices that drive the strengthening of hope.

Studies show that experiencing adverse crisis situations impacts hope, but provides learning and reflections on the meaning of life and attitudes oriented toward the future^26^ ^-^ ^28^. Temporality allowed each family member to reconstruct new meanings for the lived experiences. From this perspective, the reports about the perception of hope gave way to a positive and present meaning that was maintained and strengthened by the past experience.

The cyclical nature of the hope experience over time corroborates other studies^26^ ^-^ ^27^, which showed that hope is a dynamic resource with many facets, and that, in moments of family crisis, such as the moment of diagnosis, hope takes on an uncertain form, characterized by questions about the illness and the family’s difficulty in accepting the diagnosis. When families experience the positive impact of treatment and the illness stabilizes, uncertain hope gives way to nurturing hope, with a maintenance of the positive perspective in relation to the experienced context^26^ ^-^ ^27^.

In moments marked by uncertainty, the feelings initially expressed by the participants showed fear, anguish, and sadness, exemplifying the affective dimension that permeates the constitution of hope and hopelessness^6^ ^,^ ^29^ ^-^ ^30^.

Situations such as hospital admission were moments that meant a threat to family hope, intensifying negative feelings. Studies reveal that families, in addition to needing to face a set of negative feelings, need to deal with the “high-risk” label, which clearly points to a difference in pregnancies and birth^31^ ^-^ ^32^.

The birth of a high-risk newborn brings an intense confrontation between the imagined baby and the real baby for the family. The positive expectations about the child, built during pregnancy, are gradually deconstructed and end up becoming complex for the family, especially for mothers, who are faced with a newborn with the possibility of presenting complications and illnesses throughout life^33^ ^-^ ^34^. Added to this aspect is the context of hospital admission, which is accompanied by negative feelings^35^.

The results reveal that, in the context of hospital admission, the COVID-19 pandemic had repercussions on the lived experience. The isolation of the newborn and restrictions on entry into the neonatal ICU caused the family, especially the mother, to feel the loss of her maternal function, thus having difficulty recognizing herself as a mother^36^. This happens because, most of the time, the staff assumes care. This situation reinforced feelings such as fear and insecurity, contributing to threatening family hope^37^. This evidence allows us to infer that including the family in care, for example, through FCC, in addition to being a recommendation as it improves the neonatal prognosis, strengthens the sense of usefulness and may contribute to strengthening hope.

The moment of the pandemic concurrent with the newborn’s hospitalization contributed to the mother’s distancing from her daily routine, which can negatively impact her mental health, giving rise to feelings that favor hopelessness^38^ ^-^ ^40^.

Still within the context of the affective dimension of hope, in moments of stabilization of the neonatal risk and at the newborn’s hospital discharge, there is a reframing of the lived experience. It is identified that positive emotions such as love, relief, and happiness become resources that propel hope^6^ ^,^ ^41^.

 On the other hand, in some situations, the condition of gestational and neonatal risk did not prevent the family member from seeking to maintain positive feelings during the process. In this research, positive feelings contributed to coping with situations involving gestational and neonatal risk and to overcoming difficulties^42^ ^-^ ^43^.

It is pertinent for the family to create coping strategies, and in this research, despite the uncertain hope and the affective dimension at the discovery of the risk, oriented toward negative feelings, the family members reported reframing their emotions by sustaining their hopes through the search for spirituality, an important strategy for developing positive emotions and decision-making^6^.

The family experience in the context of gestational and neonatal risk involves negative feelings, which are overcome through religious beliefs, which constitute strategies that strengthen hope. They are strengthened through prayer practices, church attendance, and faith in God^44^.

In this context, based on the behavioral dimension^6^, among the actions that contributed to the maintenance or strengthening of hope for the participating families was religious belief, a strategy that was strengthened over time^45^. By transferring this understanding to the context of the present study, it is perceived that when family members place their religious beliefs as priorities in their lives, they may seek a justification for the context in which they find themselves, which can facilitate coping with the current situation.

The participants stated that having their spiritual needs met was important throughout the entire lived process to strengthen the care for the newborns. However, studies indicate that inquiries about religious beliefs and the valorization of spirituality, as resources for care, are still not routine in the approach of healthcare providers^41^ ^,^ ^45^ ^-^ ^46^.

The literature has revealed that most healthcare team providers are unaware of the relevance of spiritual practices for health treatment, or have a conception that such an approach is not part of their role, and there are still situations in which the healthcare provider themself has difficulty discussing the topic^45^ ^,^ ^47^ ^-^ ^48^. Within this perspective, it can be inferred that it is necessary for the nurse to recognize spirituality as a human dimension that should be included in care.

The present study demonstrated that, at the discovery of the gestational and neonatal risk, hope is constructed and perceived in the spiritual realm in an optimistic way, with the perspective of a future in which the situation passes quickly, driving family members to perform actions directed toward hope goals^45^. These feelings are understandable, considering that hope consists of a subjectively human experience that promotes spiritual well-being, as well as sustains these families by motivating them to make plans and continue care^26^. Therefore, hope is strengthened in faith and is essential to life, it enables and strengthens the person when they perceive themselves in control of their existence^49^.

The analysis of the narratives indicates that, among the strategies for maintaining hope, are the strengthening of intrapersonal skills of self-confidence and resilience, exemplifying the behavioral dimension of the model^6^. Resilience shows itself to be opportune in coping with stressful situations within the family context by contributing to considered and flexible behaviors that reframe the lived experience^50^. In most cases, these behaviors were related to learning from what was experienced in the past, since the discovery of the gestational risk.

A study conducted with families of children with chronic conditions and providers shows that open and receptive communication among family members, mutual support, and an approach to religious faith are important for the family to perform spontaneous movements of the family resilience process^51^. The results of this research allow us to state that intrapersonal relationships of self-confidence, socioemotional skills, and resilience proved to be the basis for maintaining hope behaviors.

This study presents strengths and limitations. The following are considered strengths: the inclusion of the family unit as a central component of this research and the involvement of other family members of the newborn besides the mother; the conduct of oral history interviews and subsequent construction of narratives, which aided in understanding the history and context in which the families and newborns were inserted; the rigor used in the development of this study with the detailed description of the method, which may assist in future qualitative research investigations; and the use of a theoretical framework, which allowed for a deeper analysis of the data.

As a limitation, it is recognized that the results discussed here pertain to a group of people exposed to a specific care network and that, in other locations, other additional results may be found. It is also understood that the participation of healthcare providers who assisted these families during the experience could complement the described results. Therefore, future studies that address these dimensions become pertinent. Furthermore, there remains the impetus to conduct new studies that seek to identify and understand how teaching about hope happens for students in the health area, given the importance of this theme for care.

Nevertheless, the results of this study can assist healthcare providers in their practices, in planning systemic family care, recognizing hope as an essential and dynamic family resource, identifying the barriers encountered, and implementing strategies to overcome them. Furthermore, the results contribute to the understanding that hope should be assessed in different circumstances and situations, and is related to context, behaviors, established relationships, and feelings over time. 

## Conclusion

The evidence regarding family experiences of hope in the context of pregnancy and care for the high-risk newborn contributed to reinforcing the dynamic character of hope based on different lived situations. The experiences of family hope and hopelessness are characterized through perceptions, behaviors, and feelings and are influenced by the relationships that are established, beliefs and emotions, in addition to the context in which they find themselves.

At the beginning of the experience of risk, whether gestational or neonatal, the perception of hope is fragile, evidenced by uncertainty, negative feelings, and desires for better days. Situations such as hospital admission and the COVID-19 pandemic were moments that enabled family hopelessness, increasing negative feelings regarding the lived experience. Over time and with the decrease of more critical situations, the newborn’s hospital discharge and follow-up care, the family hope experience changes and strengthens, through the maintenance of the understanding of hope, which is strengthened by past experiences. Strategies and behaviors, such as religious belief and the development of intrapersonal relationship skills, are strengthened and maintained over time, contributing to the change toward positive feelings and to the continuity of care for the newborn.

In this sense, healthcare providers must be able to identify situations of family hopelessness within the context of gestational risk and to perform concrete actions, aiming to strengthen hope experiences, such as: identifying the feelings experienced by the families and supporting positive and realistic feelings about the situation, through attentive listening and welcoming; assisting in the strengthening of hope strategies and behaviors that aim to strengthen it, such as strengthening spirituality and self-confidence and through attitudes of family encouragement, such as sharing information and empathy. There are indications that these strategies may contribute positively to family experiences and reframe the lived context.

## Data Availability

Datasets related to this article will be available upon request to the corresponding author.
