# Supplementary material for: Experiences of family hope and hopelessness in the context of pregnancy and care for high-risk newborns
Source: Rev Lat Am Enfermagem. 2026 Jul 24;34:e4834. doi: 10.1590/1518-8345.7906.4834 (PMC13405657; doi:10.1590/1518-8345.7906.4834)
Supplement: Translation

# Translation: Experiências familiares de (des)esperança no contexto da gestação e cuidados com o neonato de risco^*^

**DOI:** 10.1590/1518-8345.7906.4835

**Authors:** Bruna Camargos de Lima Ramos, Renata de Oliveira Costa, Elysângela Dittz Duarte, Zaida Borges Charepe, Patrícia Pinto Braga

**Affiliations:** 1 Universidade Federal de São João del-Rei, Curso de Enfermagem, Divinópolis, MG, Brasil.; 2 Universidade Federal de Minas Gerais, Departamento de Enfermagem, Belo Horizonte, MG, Brasil.; 3 Universidade Católica Portuguesa, Escola de Enfermagem, Lisboa, Lisboa, Portugal.

**Keywords:** Esperança, Família, Gravidez de Alto Risco, Recém-Nascido, Pesquisa em Enfermagem, Enfermagem Familiar.

## Abstract

**Objetivo:**

compreender experiências familiares de esperança, da gestação aos cuidados do neonato de risco.

**Método:**

pesquisa qualitativa, orientada pelo Modelo Teórico de Entendimento da Natureza Complexa da Esperança, que teve como cenário um ambulatório multiprofissional de atenção ao neonato de risco. Participaram 28 pessoas de 14 famílias a partir de entrevista em história oral, que permitiram a construção de narrativas familiares, as quais foram submetidas à análise temática dedutiva.

**Resultados:**

as experiências familiares de esperança se estabelecem a partir de quatro dimensões: compreensão, julgamento e interpretação acerca da esperança; aspectos afetivos e sentimentos em relação à experiência vivida; ações, comportamentos e estratégias que fortalecem a esperança familiar; influência do contexto e situações de vida. Estas são influenciadas pela temporalidade em que a família se encontra. No início da vivência do risco, a esperança mostra-se fragilizada, incerta, com sentimentos negativos e desejos de dias melhores. Com o passar do tempo, estratégias como a espiritualidade e as habilidades de relações intrapessoais, são fortalecidas e mantidas, ressignificando os sentimentos.

**Conclusão:**

compreender as experiências de (des)esperança familiar pode ajudar os profissionais no sentido de fortalecer e contribuir com uma experiência mais positiva, na presença do risco gestacional e neonatal.

## Destaques

**(1)** A esperança é um recurso essencial no contexto do risco gestacional e neonatal. **(2)** A esperança familiar é dinâmica e tem influência na temporalidade. **(3)** A experiência de esperança familiar é influenciada pelas relações, crenças e emoções. **(4)** A enfermagem deve auxiliar as famílias no fortalecimento da esperança.

## Introdução

No contexto da gestação e cuidado neonatal de risco, os familiares enfrentam situações complexas, como a confirmação do risco gestacional, internação materna ou neonatal, medos relacionados ao prognóstico e demandas frequentes por atendimentos de saúde^1^ ^-^ ^2^. O impacto dessa experiência para as famílias é multifacetado, afetando dimensões físicas, psicológicas e sociais. Gestações de alto risco frequentemente culminam em partos prematuros, baixo peso ao nascer e complicações clínicas em casos de anomalias congênitas, o que exige cuidados neonatais intensivos e pode resultar em problemas de saúde a longo prazo para o neonato^3^. 

O risco gestacional e neonatal repercute sobre o sistema familiar e é oportuno apontar que o cuidado centrado na família (CCF) tem sido associado a melhorias nos desfechos clínicos e pode auxiliar na promoção de uma cultura que valoriza as parcerias com as famílias^4^ ^-^ ^5^. Nesta perspectiva, valorizar o CCF torna-se uma estratégia oportuna para abordagem da esperança para este grupo.

Dentre os familiares, especialmente as mães relatam maior sobrecarga emocional devido às necessidades de cuidados de longa duração^4^, apresentando taxas mais altas de ansiedade, transtorno de *stress* pós-traumático e sintomas depressivos^3^ ^-^ ^4^.

Embora existam estudos sobre os impactos físicos e emocionais das gestações e cuidados neonatais de alto risco, é necessário compreender como a esperança é vivenciada, construída e ressignificada pelas famílias ao longo do tempo.

A esperança, segundo o Modelo Teórico de Entendimento da Natureza Complexa da Esperança^6^, é caracterizada como uma experiência de sentido e propósito para a vida, envolvendo expectativas de melhoria, desejos de mudança e pensamentos positivos. Este modelo oferece uma estrutura teórica para analisar as dimensões da esperança, a partir de cinco dimensões, como temporalidade, cognição, afetividade, comportamento e contexto^4^ ^-^ ^6^. Em síntese, as ideias principais destas dimensões são: a) temporal, que envolve a experiência de cada indivíduo no tempo presente, passado e futuro, em que a esperança é dirigida para o futuro, embora influenciada pelo passado e presente; b) cognitiva, relacionada ao processo pelo qual a pessoa deseja, imagina, interpreta e julga em relação à esperança, tem a percepção do desejo realista do futuro; c) afetiva, que abrange uma série de emoções e sentimentos, por vezes opostos, entre os quais há atração para o objetivo a ser atingido; d) comportamental, que relaciona-se à ação que busca o objetivo referente à esperança no âmbito físico, psicológico, social e espiritual; e) contextual, que se refere às situações de vida e ambiente que rodeiam, influenciam e constituem parte da esperança da pessoa^6^.

À semelhança de outros estudos que o abordam^7^ ^-^ ^10^, o modelo forneceu uma base para categorizar e interpretar as experiências das famílias participantes deste estudo, facilitando a identificação de padrões e temas recorrentes sob as dimensões mencionadas.

Compreender as experiências familiares de esperança pode ajudar os profissionais de saúde e enfermeiros a fortalecer e contribuir para uma vivência mais positiva diante do risco gestacional e neonatal^3^. Estratégias como escuta ativa, suporte emocional, respeito às crenças religiosas e culturais e incentivo à participação nos cuidados indicam promover a esperança familiar. Reconhecer que a experiência familiar e a (des)esperança se modificam ao longo do tempo é essencial para oferecer um cuidado mais construtivo e centrado neste grupo^3^ ^,^ ^6^. 

Mediante o exposto, buscou-se responder à seguinte questão: como se apresenta a experiência familiar de esperança, desde a gestação até os cuidados com o neonato de risco? O objetivo deste estudo consistiu em compreender experiências familiares de esperança, da gestação aos cuidados do neonato de risco.

## Método

### Delineamento do estudo

Estudo qualitativo interpretativo, orientado pelo Modelo Teórico de Entendimento da Natureza Complexa da Esperança^6^. O modelo ofereceu uma estrutura conceitual das dimensões que sustentam a esperança (temporalidade, cognição, afetividade, comportamento e contexto), apoiando o percurso teórico, metodológico e análise dos dados.

### Participantes e cenário do estudo 

Os participantes foram 28 familiares (incluindo mãe, pai, avó, tia e irmã) de 14 famílias de neonatos de risco, cujas mães tiveram a gestação avaliada como de alto risco^11^. Como o estudo objetivou compreender experiências familiares, além do cuidador principal, foram entrevistados outros integrantes das famílias, indicados pelo cuidador principal. As entrevistas com os participantes foram orientadas pelo mesmo roteiro e em momentos distintos, de forma individual e reservada. O cenário do estudo foi o domicílio dessas famílias e o ambulatório multiprofissional do Programa de Intervenção Precoce Avançado (PIPA), em Divinópolis-MG, Brasil, para atendimentos de recém-nascidos em situação de risco. O serviço acompanha neonatos e crianças de risco até completarem dois anos e realiza em média 192 atendimentos por trimestre.

Os critérios de inclusão para participação foram familiares de crianças entre seis e 12 meses durante a coleta, com histórico de risco neonatal, permitindo tempo suficiente para a experiência ser compartilhada por meio de histórias orais e estabelecer um limite temporal entre o nascimento e a entrevista. Foram excluídas famílias cujas mães tinham idade inferior a 18 anos, mulheres classificadas como alto risco na gestação devido a questões como depressão ou distúrbios psiquiátricos e famílias de mulheres que tiveram depressão ou sofrimento mental até 45 dias após o parto. Esses critérios se justificam, pois a entrevista poderia mobilizar lembranças, sofrimento ou constrangimento para as mulheres e/ou familiares.

A partir da análise de 146 prontuários do ambulatório, 60 famílias eram pré-elegíveis. Primeiramente, foi realizada uma aproximação por meio de um contato prévio por telefone, explicando o objetivo da pesquisa e, destas, 24 famílias não responderam ao convite e cinco se recusaram. 

### Coleta de dados

O contato inicial era realizado por telefone, com o cuidador principal, que nesta pesquisa foram mães (13) e pai (1). As entrevistas eram agendadas no PIPA ou no domicílio, respeitando o desejo e disponibilidade do participante. No final da entrevista era solicitada a indicação de pessoas da família que também cuidavam da criança desde o momento da descoberta do risco neonatal até o momento da entrevista. Neste momento era esclarecida a ideia de família adotada no estudo, de forma que o participante pudesse identificar pessoas envolvidas no cuidado à criança. Este processo permitiu identificar outros integrantes da família para participar posteriormente das entrevistas. Nesta pesquisa, a família é compreendida como uma estrutura relacional composta por indivíduos que possuem vínculos afetivos e/ou biológicos entre si, com comprometimento mútuo, identidade de projetos de vida e propósitos em comum^12^.

Para a coleta dos dados, realizou-se uma entrevista em história oral de caráter temático^13^, apoiada por roteiro semiestruturado, com questões referentes à caracterização da composição familiar, ao risco gestacional e neonatal e à relação interna do contexto da família. Também integravam o roteiro perguntas abertas relacionadas às experiências de esperança familiar, considerando as diferentes dimensões do modelo teórico, no período da gestação e do cuidado com o neonato de risco, e relacionadas ao padrão interrelacional familiar. O trabalho de campo foi realizado entre dezembro de 2021 e março de 2022 pela primeira autora, a qual é enfermeira, possui experiência em pesquisa qualitativa e trabalho de campo e contou com a participação de uma aluna de iniciação científica do curso de graduação em enfermagem da instituição proponente. Ambas não possuíam vínculos com os potenciais participantes ou cenários da pesquisa.

A duração média de cada entrevista em história oral temática foi de aproximadamente 45 minutos. No momento da entrevista, havia somente as entrevistadoras e os participantes.

A interrupção das entrevistas e a definição do limite de participantes aconteceram a partir da saturação teórica dos dados^14^. Para tanto, adotou-se a análise de conteúdo das entrevistas concomitantemente à coleta^14^ ^-^ ^15^. A constatação da saturação teórica dos dados ocorreu na 14.^a^ família, quando já haviam sido realizadas 28 entrevistas. 

Reconheceram-se os desafios que envolvem o processo de amostragem por saturação, portanto, durante o trabalho de análise, concomitante com a coleta dos dados, foram adotadas as seguintes estratégias para minimizar imprecisões: (a) alinhamento e estudo aprofundado do referencial adotado pelos pesquisadores previamente à sua aplicabilidade; (b) validação do dicionário de códigos elaborado a partir do referencial; (c) codificação com validação intercodificadores e reuniões para análises e consensos; (d) olhar atento para cada narrativa, identificando as semelhanças e as singularidades das experiências vividas; (e) identificação da representatividade do referencial adotado mediante as narrativas; (f) capacidade de resposta ao objetivo do estudo.

### Tratamento e análise dos dados

Após a transcrição das entrevistas, construíram-se narrativas para cada família, as quais foram submetidas à análise temática dedutiva^13^ ^,^ ^16^. Para essa finalidade, elaborou-se um dicionário de códigos, contemplando o modelo teórico^17^. Visando à validação do dicionário de códigos, uma narrativa foi analisada, simultaneamente, por três pesquisadores com domínio no referencial. Foram necessárias quatro rodadas de codificação para ajustes e alinhamentos teóricos no dicionário de códigos. Na Figura 1 é exemplificado o processo de codificação de algumas narrativas, apoiado pelas dimensões do modelo.

Seguiu-se, então, o processo de codificação de uma primeira narrativa, que foi conduzido por duas pesquisadoras de forma independente e posteriormente apresentado a uma terceira pesquisadora. Foram necessárias quatro rodadas de codificação para identificar congruência no processo. A partir de então, as narrativas foram codificadas pelas pesquisadoras e as situações de divergências ou dúvidas eram resolvidas em reunião. Esse processo garantiu a confiabilidade à codificação^16^. Após o processo de codificação, foi possível realizar agrupamentos de códigos para composição das categorias, de modo a retratar e apresentar o conteúdo da análise dedutiva das narrativas.

**Figura 1:** Exemplos de codificação de narrativas a partir do dicionário

Código	Enunciados de narrativas
**Cognição**: apreciação do indivíduo, relacionada ao processo vivido, pelo qual interpreta, julga, deseja e imagina a esperança; presença ou não de desejos realistas referentes ao futuro.	*Esperança ela nos mantém vivos. Eu acho que é isso. Eu tenho quase a certeza que se a gente não tiver esperança, não adianta viver. Então, baseado nessa pergunta eu acho que sempre tive a esperança que ia dar tudo certo* **(Avó 9).** *Esperança é acreditar e transformar. Dias melhores* **(Irmã materna 3)** *.*
**Comportamental**: ações direcionadas à esperança no âmbito físico, psicológico, social e espiritual. Estratégias que podem promover, fortalecer ou enfraquecer a esperança familiar.	*Para ter expectativa de esperança quando a gente descobriu o risco, eu e minha sogra foi lá na capela e ajoelhou, rezou. A estratégia para ter esperança foi acreditar em Deus* **(Mãe 2).** *A estratégia utilizada para ter esperança, a mais certa pra nós foi a união, estar junto mesmo, não deixar a pessoa sozinha. As vezes saí de lá, a mãe delas tava chorando, eu conversava com ela: Vai dar certo! Eu acho que a estratégia que a gente usou foi isso mesmo, foi união, garra um com o outro ali na fé, ali pra que tudo desse certo* **(Pai 13).**

Atendendo aos critérios de cientificidade, esclarecemos que, neste estudo, a credibilidade foi viabilizada a partir de análises rigorosas conduzidas simultaneamente ao processo de coleta de dados, apoiada pelas narrativas e pela construção dos instrumentos das relações de esperança. A confiabilidade esteve presente a partir da descrição detalhada do método, seguindo o *checklist Consolidated Criteria for Reporting Qualitative Research* (COREQ). A confirmabilidade foi contemplada e garantida, considerando-se a apresentação das fortalezas e limitações do estudo^18^ ^-^ ^20^.

### Aspectos éticos

O estudo foi conduzido segundo os aspectos éticos que norteiam o desenvolvimento de pesquisa com seres humanos do país, sendo aprovado pelo Comitê de Ética em Pesquisa da instituição proponente sob parecer número 5.119.278 e número do Certificado de Apresentação de Apreciação Ética (CAAE) 51866821.4.0000.5545. Todos os participantes foram orientados sobre os objetivos da pesquisa e finalizaram o processo de obtenção do consentimento por meio da assinatura do Termo de Consentimento Livre e Esclarecido (TCLE), antes do início da coleta de dados. Para garantir o anonimato, o nome de cada participante foi substituído pelo parentesco com a criança, seguido da ordem de realização das entrevistas, por exemplo: mãe 1, avô materno 1 e assim sucessivamente.

## Resultados

A análise dos dados da pesquisa permitiu sintetizar a apresentação dos familiares. Dos 28 participantes do estudo, 46,4% (13) eram mães biológicas, 21,4% (6) pais, 14,3% (4) tias, 10,7% (3) avós, 3,6% (1) madrinha, 3,6% (1) irmã. A faixa etária dos participantes variou entre 25 e 56 anos. 

Entre as condições de risco apresentadas pelos neonatos, identificamos: 15,7% (3) prematuros, 47,3% (9) prematuridade associada à gemelaridade, 10,5% (2) toxoplasmose congênita e 5,3% (1) para cada um dos seguintes riscos: rubéola, derrame pericárdico, sepse neonatal, prematuridade associada a sífilis congênita, prematuridade associada a hidronefrose neonatal e ascite. A idade das crianças no momento da pesquisa variou entre 6 meses e 1 ano. 

A história gestacional das mães foi acompanhada de risco neonatal e, dentre os riscos gestacionais, encontramos: 14,3% (2) hipertensão gestacional associada a diabetes gestacional; 14,3% (2) pré-eclâmpsia associada à gemelaridade e 5,3% (1) para cada um dos seguintes riscos: hipertensão gestacional, sífilis gestacional, toxoplasmose, toxoplasmose associada à hipertensão gestacional, hipotireoidismo, pré-eclâmpsia associada à gemelaridade e descolamento prematuro de placenta, diabetes gestacional associada a polidrâmnio, rubéola, hipertensão gestacional associada a alterações cardíacas e diabetes gestacional associada à gemelaridade. 

Na Figura 2 apresentamos os participantes por família e seu parentesco com o neonato e os riscos neonatais e gestacionais experenciados por cada família.

Os resultados da análise dedutiva serão apresentados a partir das categorias: Compreensão, julgamento e interpretação acerca da esperança; Aspectos afetivos e os sentimentos em relação à experiência vivida; Ações, comportamentos e estratégias que fortalecem a esperança familiar; Influência do contexto de vida na experiência de (des)esperança familiar.

**Figura 2:** Participantes familiares, seu parentesco com o neonato e os riscos gestacionais e neonatais. Divinópolis, MG, Brasil, 2025

Família	Participantes	Risco neonatal	Risco gestacional
F1	Pai, Mãe	Prematuridade	Síndrome HELLP* e diabetes
F2	Mãe, Avó, Madrinha	Sífilis congênita; prematuridade	Sífilis
F3	Mãe, Tia	Toxoplasmose congênita	Toxoplasmose
F4	Mãe, Irmã	Toxoplasmose congênita	Toxoplasmose e hipertensão
F5	Mãe, Tia	Prematuridade	Pré-eclâmpsia e gemelaridade
F6	Pai	Prematuridade, trigemelaridade	Hipertensão, trigemelaridade e diabetes
F7	Mãe, Tia	Prematuridade	Hipertensão e diabetes
F8	Mãe, Pai	Prematuridade	Hipotireoidismo
F9	Mãe, Avó	Prematuridade; gemelaridade	Pré-eclâmpsia, gemelaridade, descolamento de placenta, COVID-19
F10	Mãe, Pai	Prematuridade	Diabetes, polidrâmnio
F11	Mãe, Tia	Derrame pericárdico	Hipertensão e alterações cardíacas
F12	Mãe ,Pai	Rubéola congênita	Rubéola
F13	Mãe, Pai	Prematuridade	Diabetes gestacional e gemelaridade
F14	Mãe, Avô	Sepse neonatal	Hipertensão

*HELLP = *Hemolysis, Elevated Liver enzymes, Low Platelets*

### Compreensão, julgamento e interpretação acerca da esperança

De forma geral, os familiares possuem diferentes compreensões sobre a palavra “Esperança”. Todos os participantes relataram que a esperança esteve presente durante toda a trajetória de cuidado à criança, desde a descoberta da gestação de risco até os dias atuais. A compreensão sobre esperança se modificava e/ou se fortalecia a partir da experiência vivida. Desde a descoberta do risco gestacional e neonatal, assim como após o nascimento do neonato, ela foi evidenciada como um processo de crença em um futuro melhor, no desejo e espera de dias melhores.

*Quando descobri o risco eu tinha esperança daquilo tudo mudar. Que dias melhores poderia vir.*

(Mãe 10)

*Quando a gente se toca que o bebê nasceu com o risco, aí a gente entende o que é esperança. Sempre pensando em dias melhores.*

(Avó 9)

*Eu tinha muita esperança ali era de primeiramente o parto ocorrer bem, né, com a mãe, eu já tinha essa ciência que eram prematuras, né, que poderiam acontecer alguma coisa com elas. Então eu acho que é isso, é esperança de dar tudo certo, elas nascer bem, nascer saudável, por mais que a gente passou essa barra toda no CTI, eu sempre tive essa esperança de dar tudo certo.*

(Pai 13)

A análise aponta que, ao longo do tempo, a esperança é interpretada como uma experiência positiva que pode ter sido fortalecida pela experiência passada, por exemplo, de descoberta do risco gestacional, remetendo à ideia de perseverança e oportunidade de reacreditar na vida.

*Esperança é tudo aquilo que eu vivi. Desde que eu descobri a minha gestação, que elas poderiam vir prematuras. E agora eu percebo o quanto foi importante a esperança pra mim*

(Mãe 13)

*Esperança é aquilo que eu acreditei. É tentar achar a luz realmente lá de dentro, acreditar que tudo iria mudar. E, olha, mudou.*

(Irmã paterna 4)

*É uma oportunidade nova de estar vivendo, todos os dias. Elas (filhas gemelares) me deram a oportunidade de reacreditar na vida. Lutar pela minha família.*

(Pai 6)

### Aspectos afetivos e os sentimentos em relação à experiência vivida

A experiência familiar na trajetória dos riscos gestacionais e neonatais foi construída a partir de sentimentos e emoções ambíguos e contraditórios, favorecendo ou não a manutenção da esperança e que se modificaram ao longo do tempo. A análise permite evidenciar que houve uma ressignificação da situação vivida, a partir da temporalidade, e isso fez com que os familiares retratassem uma modificação dos seus sentimentos. 

No contexto da descoberta do risco que envolveu a gestação e o neonato, os sentimentos foram relatados inicialmente como negativos, estando presentes o medo, ansiedade, apreensão, desespero, tristeza, surpresa, desconfiança, culpa, raiva.

*Ao descobrir o risco dela eu fiquei muito chateada e muito apreensiva.*

(Mãe 1)

*Acho que ansiedade tomou conta de mim. Eu fiquei muito ansiosa. Isso fez com que a minha esperança ficasse abalada.*

(Mãe 8)

*Não consigo falar de outra coisa que não seja medo. Até quando ela nasceu eu tinha medo, tava tudo estranho.*

(Pai 12)

*Nossa, eu me senti muito culpada. Saber que teria, poderia ter, um risco por causa de mim.*

(Mãe 4)

Apesar das incertezas, após o nascimento do neonato, sentimentos como felicidade, amor, confiança e alívio, foram relatados. Para a maioria dos familiares, estes sentimentos se mantêm nos dias atuais.

*Olha, depois que a tempestade passou, que eu vi que ela nasceu bem, o alívio, a felicidade, foi tomando conta de mim.*

(Mãe 14)

*O que mais me domina até o hoje é o sentimento de felicidade, né, com certeza. Quando vi que ele ficou bem depois que saiu da Unidade de Terapia Intensiva (UTI), foi muito choro de alegria. Hoje é isso, só alegria.*

(Avô materno 14)

Alguns familiares relatam uma manutenção de sentimentos positivos, como felicidade e amor, durante toda a experiência de cuidados com o neonato, desde a descoberta do risco gestacional e/ou neonatal.

*Quando eu descobri era só amor. Senti amor o tempo todo. Até hoje.*

(Mãe 10)

*Acho que apesar de tudo, o sentimento que mais prevalece é felicidade mesmo. Isso me fez ter esperança.*

(Pai 10)

Ações, comportamentos e estratégias que fortalecem a esperança familiar

A busca de estratégias e fontes fortalecedoras de esperança, desde a gestação e após o nascimento do neonato, envolveu principalmente as práticas de espiritualidade e habilidades de desenvolvimento das relações intrapessoais. Estas estratégias estiveram presentes desde a descoberta do risco e, ao longo do tempo, foram fortalecidas.

Os familiares utilizam estratégias que visam fortalecer o âmbito espiritual e religioso, por intermédio da fé em Deus, por meio da oração e visita à igreja. Estas estratégias estiveram presentes desde a descoberta da gestação de risco e vêm sendo fortalecidas até os dias atuais.

*O que eu usei para ter esperança foi Jesus. Eu orei muito. Foi Deus na frente.*

(Mãe 1)

*Usei Deus pra combater isso, e aí a esperança vinha. E até hoje a fé em Deus prevalece, com certeza.*

(Pai 1)

*Sempre tive muita fé, muita fé, sabe? Desde que descobri, descobri o risco. Rezei muito, ia na missa sempre, e hoje mais ainda, eu sou grata.*

(Mãe 4)

*Estratégias que me ajudaram a ter esperança foi fortalecer a minha espiritualidade, procurar mais a igreja, isso me ajuda até hoje.*

(Mãe 11)

Relações intrapessoais de envolvimento consigo mesmo também foram consideradas habilidades desenvolvidas para se ter esperança. Os familiares referem ter sido seu próprio ponto de apoio e de ação, principalmente a mãe, na experiência de gestação e nascimento do neonato de risco.

*Eu tive que vencer, fui muito forte ali no momento pra conseguir vencer.*

(Mãe 6)

*A gente tem que ir levando a gravidez até onde ela aguentar, e vai dar tudo certo. Aí foi onde eu fui assim, sabe, tendo mais esperança.*

(Mãe 11)

A resiliência e controle de emoções, autoconfiança para continuar cuidando do neonato foram identificados para buscar o objetivo referente à manutenção da esperança nos dias atuais.

*É o pensamento positivo… é, tentar controlar mesmo meus pensamentos, sabe? Isso me ajuda muito.*

(Mãe 4)

*Depois que passamos por tudo, eu falo que aprendi a ser resiliente, em tudo na vida. Pelo menos isso tudo serviu de aprendizado pra minha vida pessoal também.*

(Tia materna 5)

*Depois do que passei, depois de todo estresse, de tanto chorar, e acho que consigo passar por tudo agora. Fiquei mais forte, mais resiliente.*

(Pai 10)

### Influência do contexto e situações de vida na experiência de (des)esperança familiar

A experiência de esperança foi influenciada pelo contexto vivido pelos familiares. A análise permitiu identificar que a internação do neonato, associada à pandemia da COVID-19, marcou as experiências de esperança. A internação do recém-nascido, devido ao risco, foi descrita como um período que ameaçou a esperança e até mesmo gerou desesperança. Neste momento, as famílias relatam que o contato com o neonato foi adiado e que foi difícil retornar para casa sem a presença do(a)filho(a).

*Elas (filhas gemelares) ficaram internadas, né, só isso já foi ruim. É muito ruim a gente ter que sair de lá e deixar elas. Às vezes eu saía de lá desacreditado…*

(Pai 13)

*A internação fez com que minha esperança fosse ameaçada. Ver eles (filhos gemelares) internados sem saber quando iriam sair, e se iriam sair... eu só chorava e mais nada.*

(Mãe 9)

O contexto da pandemia influenciou negativamente as experiências familiares, restringindo e adiando o contato com o neonato após o nascimento, aumentando o sentimento de insegurança e preocupação.

*Só essa pandemia já é ruim né. A gente não consegue fazer as coisas direito… eu não conseguia ver meu filho muito e nem pegar, porque era tudo muito frágil. Eu me senti impotente.*

(Pai 1)

*A gente chegou lá, já não bastava a preocupação do caso delas, tinha o COVID pra fazer a gente ficar desesperançoso, né? Ambiente de hospital já não é bom, com a pandemia então...*

(Pai 10)

A análise das narrativas evidenciou que as experiências durante a gestação, o nascimento, após o parto e nos cuidados neonatais, incluindo os dias atuais com a criança, contribuíram para que as famílias ressignificassem e construíssem sua “experiência de esperança”, apesar de terem vivenciado momentos de desesperança. Desta forma, os resultados apresentam estas experiências e suas modificações ao longo do tempo. 

Na Figura 3, é apresentada uma síntese dos principais resultados desta investigação e evidencia os elementos que mantiveram, fortaleceram ou ameaçaram a esperança em um percurso marcado por mudanças e oscilações referentes à (des)esperança.

**Figura 3:** (Des)Esperança familiar ao longo da gestação e cuidados neonatais de risco. Divinópolis, MG, Brasil, 2025

## Discussão

Os resultados evidenciaram que as experiências familiares de esperança se estabelecem a partir da compreensão de crença em um futuro melhor, emoções ambíguas, comportamentos que buscam fortalecer a esperança e ao contexto de vida, sendo estes fatores que se modificam ao longo do tempo. 

A análise das narrativas permite afirmar que as histórias das famílias apresentam algumas semelhanças, como o tipo de risco gestacional ou neonatal e a estrutura familiar. A experiência de esperança de cada familiar foi singular, influenciada pelo ambiente em que estão inseridos, refletindo a dimensão contextual disposta no modelo adotado^6^.

Nesta pesquisa, ficou evidente que os riscos gestacionais maternos foram acompanhados de riscos neonatais. Isto reitera a relevância de identificar o perfil obstétrico e risco gestacional materno de todos os neonatos, favorecendo o prognóstico, reduzindo as chances de morbimortalidade, de internação hospitalar e comprometimento da sua saúde ao longo da vida^21^ ^-^ ^22^.

O risco gestacional (hipertensão) e neonatal (prematuridade) prevalentes nesta investigação são semelhantes aos de outras realidades nacionais e internacionais^23^ ^-^ ^24^. Isso reafirma a importância de um olhar atento acerca dos fatores que influenciam e predispõem o grupo materno-infantil a desfechos negativos. 

Os relatos dos familiares evidenciaram que a crença e desejo dos participantes em dias melhores e as expectativas positivas relativas ao futuro foram fortalecidas e mantidas ao longo do tempo, a partir de todo o contexto vivido. Nesta perspectiva, podemos inferir que a cognição^6^, expressa na compreensão e julgamentos direcionados ao futuro, favoreceu uma construção realista, apesar de o futuro materno-infantil ser marcado por incertezas.

O momento de estabilização do neonato, após a família passar por momentos como a internação, que ameaçaram a esperança, colabora para intensificar a expectativa de que o risco vai ser superado^25^. Assim, apesar da experiência do risco, a esperança foi mantida, fortalecendo uma crença positiva. Os dados parecem indicar que os momentos de crise, como a descoberta do risco ou internação neonatal, funcionam, dependendo das interações e apoio estabelecidos, como dispositivos que impulsionam o fortalecimento da esperança. 

Estudos mostram que vivenciar situações adversas de crise impacta a esperança, mas proporciona um aprendizado e reflexões sobre o sentido da vida e atitudes orientadas para o futuro^26^ ^-^ ^28^. A temporalidade permitiu que cada familiar reconstruísse novos sentidos para as experiências vividas. Nesta perspectiva, os relatos sobre a percepção da esperança deram lugar a um sentido positivo e presente que foi mantido e fortalecido pela experiência passada.

O caráter cíclico da experiência de esperança ao longo do tempo corrobora outros estudos^26^ ^-^ ^27^, que evidenciaram que a esperança é um recurso dinâmico e com muitas faces, sendo que, em momentos de crise familiar, como o momento do diagnóstico, a esperança assume uma forma incerta, caracterizada por questionamentos sobre a doença e a dificuldade da família em aceitar o diagnóstico. Quando as famílias experienciam o impacto positivo do tratamento e a doença se estabiliza, a esperança incerta dá lugar a uma esperança cuidativa, existindo uma manutenção da perspectiva positiva em relação ao contexto vivenciado^26^ ^-^ ^27^.

Em momentos marcados por incerteza, os sentimentos inicialmente expressos pelos participantes evidenciaram medo, angústia e tristeza, exemplificando a dimensão afetiva que perpassa a constituição da (des)esperança^6^ ^,^ ^29^ ^-^ ^30^. 

Situações como a internação hospitalar foram momentos que significaram ameaça à esperança familiar, intensificaram os sentimentos negativos. Estudos revelam que as famílias, além de precisar enfrentar um conjunto de sentimentos negativos, precisam lidar com o “rótulo” alto risco, que claramente aponta para uma diferença nas gestações e nascimento^31^ ^-^ ^32^.

O nascimento de um recém-nascido de risco traz para a família um confronto intenso entre o bebê imaginário e o bebê real. As expectativas positivas sobre o filho, construídas na gestação, são desconstruídas gradualmente e acabam se tornando complexas para a família, principalmente para as mães, que se deparam com um recém-nascido com possibilidade de apresentar complicações e adoecimentos ao longo da vida^33^ ^-^ ^34^. Somado a este aspecto, está presente o contexto da internação hospitalar, que é acompanhado de sentimentos negativos^35^. 

Os resultados revelam que, no contexto da internação hospitalar, a pandemia da COVID-19 trouxe repercussão para a experiência vivida. O isolamento do recém-nascido e restrições de entrada na UTI neonatal fizeram com que a família, principalmente a mãe, sentisse a perda de sua função materna, tendo, assim, dificuldade de reconhecer-se como mãe^36^. Isso acontece, pois, na maioria das vezes, a equipe assume os cuidados. Esta situação reforçou sentimentos como o medo e a insegurança, contribuindo para ameaçar a esperança familiar^37^. Estas evidências permitem inferir que incluir a família nos cuidados, como, por exemplo, a partir do CCF, além de ser uma recomendação, pois melhora o prognóstico neonatal, fortalece o senso de utilidade e poderá contribuir para o fortalecimento da esperança.

O momento de pandemia concomitante à internação do recém-nascido contribuiu para o distanciamento materno do seu cotidiano, o que pode impactar negativamente em sua saúde mental, dando origem a sentimentos que favorecem a desesperança^38^ ^-^ ^40^.

Ainda no contexto da dimensão afetiva da esperança, em momentos de estabilização do risco neonatal e na alta hospitalar do neonato, existe uma ressignificação da experiência vivida. Identifica-se que emoções positivas como amor, alívio e felicidade tornam-se recursos propulsores da esperança^6^ ^,^ ^41^.

 Por outro lado, em algumas situações, a condição do risco gestacional e neonatal não impediu que o familiar buscasse manter sentimentos positivos durante o processo. Nesta pesquisa, sentimentos positivos contribuíram para o enfrentamento de situações envolvendo o risco gestacional e neonatal e para superar as dificuldades^42^ ^-^ ^43^. 

É oportuno que a família crie estratégias de enfrentamento e, nesta pesquisa, apesar da esperança incerta e da dimensão afetiva na descoberta do risco, voltada para sentimentos negativos, os familiares referiram ressignificar suas emoções ao sustentarem suas esperanças por meio da busca da espiritualidade, uma importante estratégia para a elaboração de emoções positivas e tomadas de decisão^6^. 

A experiência familiar no contexto do risco gestacional e neonatal envolve sentimentos negativos, que são superados por intermédio das crenças religiosas, que se constituem em estratégias fortalecedoras da esperança. Elas são fortalecidas por meio de práticas de oração, frequência à igreja e fé em Deus^44^. 

Nesse contexto, a partir da dimensão comportamental^6^, dentre as ações que contribuíram para a manutenção ou fortalecimento da esperança das famílias participantes, foi a crença religiosa, uma estratégia que foi fortalecida ao longo do tempo^45^. Ao deslocar esse entendimento para o contexto do presente estudo, percebe-se que os familiares, ao colocarem suas crenças religiosas como prioridades em sua vida, podem buscar uma justificativa para o contexto em que se encontram, o que pode facilitar o enfrentamento da situação atual. 

Os participantes afirmaram ser importante o atendimento de suas necessidades espirituais, durante todo o processo vivenciado, para fortalecer os cuidados com os neonatos. Entretanto, estudos apontam que os questionamentos acerca das crenças religiosas e valorização da espiritualidade, como recursos para o cuidado, ainda não são rotineiros na abordagem dos profissionais de saúde^41^ ^,^ ^45^ ^-^ ^46^.

A literatura tem revelado que a maioria dos profissionais das equipes de saúde não conhece a relevância das práticas espirituais para o tratamento em saúde, ou tem uma concepção de que tal abordagem não faz parte do seu papel, e ainda há situações nas quais o próprio profissional de saúde tem dificuldade em discutir sobre a temática^45^ ^,^ ^47^ ^-^ ^48^. Dentro dessa perspectiva, inferimos que, faz-se necessário o reconhecimento pelo enfermeiro de que a espiritualidade é uma dimensão humana, que deve fazer parte da assistência.

Neste estudo identificamos que, na descoberta do risco gestacional e neonatal, a esperança é construída e percebida no âmbito espiritual de forma otimista, com a perspectiva de um futuro em que a situação passe logo, impulsionando os familiares a realizarem ações direcionadas para objetivos de esperança^45^. Esses sentimentos são compreensíveis, considerando-se que a esperança consiste em uma experiência subjetivamente humana, que promove o bem-estar espiritual, bem como sustenta estas famílias ao motivá-las a elaborar planos e continuar os cuidados^26^. Portanto, a esperança se fortalece na fé e é essencial à vida, habilita e fortalece a pessoa quando se percebe no controle de sua existência^49^.

A análise das narrativas indica que, dentre as estratégias para a manutenção da esperança, estão o fortalecimento de habilidades intrapessoais de autoconfiança e resiliência, exemplificando a dimensão comportamental do modelo^6^. A resiliência mostra-se oportuna no enfrentamento de situações de estresse dentro do contexto familiar por contribuir com comportamentos ponderados e flexíveis, que ressignificam a experiência vivida^50^. Na maioria dos casos, esses comportamentos estavam relacionados ao aprendizado do que foi vivenciado no passado, desde a descoberta do risco gestacional.

Estudo realizado com famílias de crianças com condições crônicas e profissionais mostra que a comunicação aberta e receptiva entre os familiares, suporte mútuo e uma aproximação com a fé religiosa são importantes para que a família realize movimentos espontâneos do processo de resiliência familiar^51^. Os resultados desta pesquisa permitem afirmar que relações intrapessoais de autoconfiança, habilidades socioemocionais e resiliência mostraram-se como base para a manutenção de comportamentos de esperança.

Este estudo apresenta fortalezas e limitações. São considerados fortalezas: a inclusão da unidade familiar como componente central desta pesquisa e o envolvimento de outros membros da família do neonato, além da mãe; a realização de entrevistas em história oral e posterior construção de narrativas, o que auxiliou na compreensão da história e do contexto no qual as famílias e neonatos estavam inseridos; o rigor utilizado no desenvolvimento deste estudo com a descrição detalhada do método, que poderá auxiliar em futuras pesquisas de investigação qualitativa e a utilização de um referencial teórico, que permitiu uma análise mais profunda dos dados. 

Como limitação, reconhece-se que os resultados aqui tratados são referentes a um grupo de pessoas que está exposto a uma rede de atenção específica e que, em outras localidades, outros resultados adicionais podem ser encontrados. Compreende-se ainda que a participação de profissionais da saúde que assistiram a estas famílias durante a experiência poderia complementar os resultados descritos. Assim, tornam-se oportunos estudos futuros que se ocupem destas dimensões. Além disso, fica a inquietação de realizar novos estudos que busquem identificar e compreender como acontece o ensino sobre a esperança para estudantes da área da saúde, tendo em vista a importância desse tema para o cuidado.

Todavia, os resultados deste estudo podem auxiliar profissionais de saúde em suas práticas, no planejamento de um cuidado familiar sistêmico, reconhecendo a esperança como um recurso familiar essencial e dinâmico, identificando as barreiras encontradas e implementando estratégias para superá-las. Além disso, os resultados contribuem com o entendimento de que a esperança deve ser avaliada em diferentes conjunturas e situações, e está relacionada ao contexto, comportamentos, relações estabelecidas e sentimentos ao longo do tempo. 

## Conclusão

As evidências acerca das experiências familiares de esperança no contexto da gestação e cuidados ao neonato de risco contribuíram para reforçar o caráter dinâmico da esperança a partir de diferentes situações vividas. As experiências de (de)desesperança familiar se caracterizam por meio de percepções, comportamentos e sentimentos e são influenciadas pelas relações que se estabelecem, crenças e emoções, além do contexto em que se encontram. 

No início da vivência do risco, seja ele gestacional ou neonatal, a percepção sobre esperança se encontra fragilizada, evidenciada pela incerteza, sentimentos negativos e desejos de dias melhores. Situações como a internação hospitalar e a pandemia da COVID-19 foram momentos que possibilitaram a desesperança familiar, aumentando sentimentos negativos em relação ao vivido. Com o passar do tempo e a diminuição das situações mais críticas, a alta hospitalar do recém-nascido e seguimento do cuidado, a experiência de esperança familiar vai se modificando e se fortalecendo, por meio da manutenção sobre a compreensão da esperança, sendo esta fortalecida pelas experiências passadas. Estratégias e comportamentos, como a crença religiosa e desenvolvimento de habilidades de relações intrapessoais, são fortalecidos e mantidos ao longo do tempo, contribuindo para a mudança de sentimentos positivos e para a continuidade dos cuidados ao neonato. 

Nesse sentido, é necessário que os profissionais sejam capazes de identificar situações de desesperança familiar em meio ao contexto do risco gestacional e realizar ações concretas, no sentido de fortalecer as experiências de esperança, como: identificar os sentimentos vivenciados pelas famílias e apoiar sentimentos positivos e realistas sobre a situação, pela escuta atenta e acolhimento; auxiliar no fortalecimento de estratégias e comportamentos de esperança que visam fortalecê-la, como fortalecimento da espiritualidade e da autoconfiança e por meio de atitudes de encorajamento familiar, como compartilhamento de informações e empatia. Há indicativos de que estas estratégias poderão contribuir de forma positiva com as experiências familiares e ressignificar o contexto vivido.

## Data Availability

Os conjuntos de dados relacionados a este artigo estarão disponíveis mediante solicitação ao autor correspondente.

# Translation: Experiencias familiares de (des)esperanza en el contexto del embarazo y el cuidado del recién nacido en riesgo^*^

**DOI:** 10.1590/1518-8345.7906.4833

**Authors:** Bruna Camargos de Lima Ramos, Renata de Oliveira Costa, Elysângela Dittz Duarte, Zaida Borges Charepe, Patrícia Pinto Braga

**Affiliations:** 1 Universidade Federal de São João del-Rei, Curso de Enfermagem, Divinópolis, MG, Brasil.; 2 Universidade Federal de Minas Gerais, Departamento de Enfermagem, Belo Horizonte, MG, Brasil.; 3 Universidade Católica Portuguesa, Escola de Enfermagem, Lisboa, Lisboa, Portugal.

**Keywords:** Esperanza, Família, Embarazo de Alto Riesgo, Recién Nacido, Investigación en Enfermería, Enfermería de la Familia.

## Abstract

**Objetivo:**

comprender las experiencias familiares llenas de esperanza, desde el embarazo hasta el cuidado del recién nacido en riesgo.

**Método:**

investigación cualitativa, guiada por el Marco Teórico de Comprender la Naturaleza Compleja de la Esperanza, que tuvo lugar en una clínica ambulatoria multiprofesional para el cuidado de recién nacidos en riesgo. 28 personas de 14 familias participaron en una entrevista de historia oral, que permitió construir narrativas familiares, que fueron sometidas a análisis temático deductivo.

**Resultados:**

las experiencias familiares de esperanza se establecen a partir de cuatro dimensiones: comprensión, juicio e interpretación de la esperanza; aspectos afectivos y sentimientos en relación con la experiencia vivida; acciones, comportamientos y estrategias que fortalezcan la esperanza familiar; influencia del contexto y de las situaciones de la vida. Estos están influenciados por la temporalidad en la que se encuentra la familia. Al principio de la experiencia del riesgo, la esperanza se debilita, es incierta, con sentimientos negativos y deseos de días mejores. Con el tiempo, estrategias como la espiritualidad y las habilidades de relaciones intrapersonales se fortalecen y mantienen, resignificando sentimientos.

**Conclusión:**

comprender las experiencias de la (des)esperanza familiar puede ayudar a los profesionales a fortalecer y contribuir a una experiencia más positiva en presencia de riesgo gestacional y neonatal.

## Destacados

**(1)** La esperanza es un recurso esencial en el contexto del riesgo gestacional y neonatal. **(2)** La esperanza familiar es dinámica e influye en la temporalidad. **(3)** La experiencia de la esperanza familiar es influida por las relaciones, creencias y emociones. **(4)** La enfermería debe ayudar a las familias a fortalecer la esperanza.

## Introducción

En el contexto del embarazo y la atención neonatal de alto riesgo, los familiares se enfrentan a situaciones complejas, como la confirmación del riesgo gestacional, hospitalización materna o neonatal, temores relacionados con el pronóstico y demandas frecuentes de atención sanitaria^1^ ^-^ ^2^. El impacto de esta experiencia en las familias es multifacético, afectando a dimensiones físicas, psicológicas y sociales. Los embarazos de alto riesgo suelen culminar en partos prematuros, bajo peso al nacer y complicaciones clínicas en casos de anomalías congénitas, que requieren cuidados neonatales intensivos y pueden provocar problemas de salud a largo plazo para el recién nacido^3^.

El riesgo gestacional y neonatal tiene repercusiones en el sistema familiar y es apropiado señalar que la atención centrada en la familia (ACF) se ha asociado con mejoras en los resultados clínicos y puede ayudar a promover una cultura que valora las asociaciones con las familias^4^ ^-^ ^5^. Desde esta perspectiva, valorar la ACF se convierte en una estrategia oportuna para acercarse a la esperanza para este grupo.

Entre los familiares, especialmente las madres, informan de una mayor sobrecarga emocional debido a las necesidades de cuidados a largo plazo^4^, presentando tasas más altas de ansiedad, trastorno de estrés postraumático y síntomas depresivos^3^ ^-^ ^4^.

Aunque existen estudios sobre los impactos físicos y emocionales de los embarazos de alto riesgo y la atención neonatal, es necesario entender cómo se experimenta, construye y representa la esperanza por las familias a lo largo del tiempo.

La esperanza, según el Marco Teórico de Comprender la Naturaleza Compleja de la Esperanza^6^, se caracteriza como una experiencia de sentido y propósito para la vida, que implica expectativas de mejora, deseos de cambio y pensamientos positivos. Este modelo ofrece una estructura teórica para analizar las dimensiones de la esperanza, basada en 6 dimensiones, como la temporalidad, la cognición, la afectividad, el comportamiento y el contexto^4^ ^-^ ^6^. En resumen, las ideas principales de estas dimensiones son: a) temporal, que implica la experiencia de cada individuo en el presente, pasado y futuro, en la que la esperanza se dirige hacia el futuro, aunque influye por el pasado y el presente; b) cognitivo, relacionado con el proceso por el cual la persona desea, imagina, interpreta y juzga en relación con la esperanza, tiene la percepción del deseo realista para el futuro; c) afectiva, que abarca una serie de emociones y sentimientos, a veces opuestos, entre los cuales hay atracción hacia el objetivo a alcanzar; d) conductual, que está relacionada con la acción que busca el objetivo de la esperanza en los ámbitos físico, psicológico, social y espiritual; e) contextual, que se refiere a las situaciones de vida y el entorno que rodean, influyen y constituyen parte de la esperanza de la persona^6^.

Al igual que otros estudios que lo abordan^7^ ^-^ ^10^, el modelo proporcionó una base para categorizar e interpretar las experiencias de las familias participantes en este estudio, facilitando la identificación de patrones y temas recurrentes bajo las dimensiones mencionadas.

Comprender las experiencias familiares de esperanza puede ayudar a los profesionales de la salud y enfermeros a fortalecer y contribuir a una experiencia más positiva frente al riesgo gestacional y neonatal^3^. Estrategias como la escucha activa, el apoyo emocional, el respeto por las creencias religiosas y culturales, y el fomento de la participación en el cuidado indican que se promueve la esperanza familiar. Reconocer que la experiencia familiar y la (des)esperanza cambian con el tiempo es esencial para ofrecer una atención más constructiva y centrada a este grupo^3^ ^,^ ^6^.

Basándonos en lo anterior, buscamos responder a la siguiente pregunta: ¿cómo se presenta la experiencia familiar de esperanza, desde el embarazo hasta el cuidado del recién nacido, en riesgo? El objetivo de este estudio fue comprender las experiencias familiares llenas de esperanza, desde el embarazo hasta el cuidado del recién nacido en riesgo.

## Método

### Diseño del estudio

Este es un estudio cualitativo interpretativo, guiado por el Marco Teórico de Comprender la Naturaleza Compleja de la Esperanza^6^. El modelo ofrecía un marco conceptual de las dimensiones que sostienen la esperanza (temporalidad, cognición, afectividad, comportamiento y contexto), apoyando el camino teórico, metodológico y de análisis de datos.

### Participantes y escenario del estudio 

Los participantes eran 28 familiares (incluyendo madre, padre, abuela, tía y hermana) de 14 familias de recién nacidos de alto riesgo, cuyas madres tuvieron sus embarazos evaluados como de alto riesgo^11^. Como el estudio tenía como objetivo comprender las experiencias familiares, además del cuidador principal, se entrevistaron a otros miembros de la familia, indicados por el cuidador principal. Las entrevistas con los participantes se guiaron por el mismo guion y en diferentes momentos, tanto de forma individual como privada. El entorno del estudio fue el hogar de estas familias y de la clínica ambulatoria multiprofesional del Programa Avanzado de Intervención Temprana (PIPA), en Divinópolis-MG, Brasil, para el cuidado de recién nacidos en riesgo. El servicio sigue a neonatos y niños en riesgo hasta que cumplen 2 años y realiza una media de 192 consultas por trimestre.

Los criterios de inclusión para la participación fueron miembros de la familia de niños entre 6 y 12 meses durante la recogida de datos, con antecedentes de riesgo neonatal, permitiendo tiempo suficiente para compartir la experiencia a través de historias orales y estableciendo un límite de tiempo entre el nacimiento y la entrevista. Se excluyeron las familias cuyas madres tenían menos de 18 años, las mujeres clasificadas como de alto riesgo durante el embarazo debido a problemas como la depresión o trastornos psiquiátricos, y las familias de mujeres que sufrieron depresión o malestar mental hasta 45 días después del parto. Estos criterios están justificados, ya que la entrevista podría movilizar recuerdos, sufrimiento o vergüenza para las mujeres y/o familiares.

Según el análisis de 146 historiales médicos de la clínica ambulatoria, 60 familias eran preelegibles. Primero, se estableció un contacto previo por teléfono, explicando el objetivo de la investigación y, de estas, 24 familias no respondieron a la invitación y cinco se negaron. 

### Recogida de datos

El contacto inicial se realizó por teléfono con el cuidador principal, que en este estudio eran madres (13) y padre (1). Las entrevistas se programaron en PIPA o en casa, respetando el deseo y la disponibilidad del participante. Al final de la entrevista, se solicitó la indicación de los familiares que también cuidaron del niño desde el momento del descubrimiento del riesgo neonatal hasta el momento de la entrevista. En este punto, se aclaró la idea de la familia adoptada en el estudio, para que el participante pudiera identificar a las personas involucradas en el cuidado del niño. Este proceso permitió identificar a otros miembros de la familia para participar posteriormente en las entrevistas. En esta investigación, la familia se entiende como una estructura relacional compuesta por individuos que mantienen vínculos afectivos y/o biológicos entre sí, con compromiso mutuo, identidad de proyectos vitales y propósitos comunes^12^.

Para la recogida de datos, se realizó una entrevista de historia oral con un personaje temático^13^, apoyada por un guion semiestructurado, con preguntas relacionadas con la caracterización de la composición familiar, el riesgo gestacional y neonatal y la relación interna del contexto familiar. También formaban parte del guion preguntas abiertas relacionadas con las experiencias de la esperanza familiar, considerando las diferentes dimensiones del modelo teórico, en el periodo del embarazo y el cuidado del recién nacido en riesgo, y relacionadas con el patrón interrelacional familiar. El trabajo de campo se llevó a cabo entre diciembre de 2021 y marzo de 2022 por el primer autor, que es enfermero, tiene experiencia en investigación cualitativa y trabajo de campo, y contó con la participación de un estudiante de iniciación científica del curso de enfermería de grado en la institución proponente. Ambos no tenían vínculos con los posibles participantes ni con escenarios de investigación.

La duración media de cada entrevista de historia oral temática era de aproximadamente cuarenta y cinco minutos. En el momento de la entrevista, solo estaban los entrevistadores y los participantes.

La interrupción de las entrevistas y la definición del límite de participantes se produjeron en función de la saturación teórica de los datos^14^. Para ello, el análisis de contenido de las entrevistas se adoptó de forma simultánea con la recopilación de datos^14^ ^-^ ^15^. La constatación de la saturación teórica de los datos ocurrió en la 14ª familia, cuando ya se habían realizado 28 entrevistas. 

Se reconocieron los desafíos implicados en el proceso de muestreo por saturación, por lo tanto, durante el trabajo de análisis, junto con la recogida de datos, se adoptaron las siguientes estrategias para minimizar las inexactitudes: (a) alineación y estudio en profundidad del marco adoptado por los investigadores antes de su aplicabilidad; (b) validación del diccionario de código preparado a partir de la referencia; (c) codificación con validación intercodificadora y reuniones para análisis y consenso; (d) un análisis cuidadoso de cada narrativa, identificando las similitudes y singularidades de las experiencias vividas; (e) identificación de la representatividad del marco adoptado a través de las narrativas; (f) capacidad de respuesta al objetivo del estudio.

### Procesamiento y análisis de datos

Tras la transcripción de las entrevistas, se construyeron narrativas para cada familia, que se sometieron a un análisis temático deductivo^13^ ^,^ ^16^. Para ello, se elaboró un diccionario de códigos, contemplando el modelo teórico^17^. Con el objetivo de validar el diccionario de códigos, una narrativa fue analizada simultáneamente por tres investigadores con dominio del referencial. Fueron necesarias 4 rondas de codificación para ajustes teóricos y alineaciones en el diccionario de códigos. La Figura 1 ejemplifica el proceso de codificación de algunas narrativas, respaldadas por las dimensiones del modelo.

**Figura 1:** Ejemplos de codificación de narrativas del diccionario

Código	Enunciados de narrativas
**Cognición**: apreciación del individuo, relacionada con el proceso vivido, a través de la cual interpreta, juzga, desea e imagina la esperanza; presencia o ausencia de deseos realistas respecto al futuro.	*Esperanza, eso nos mantiene vivos. Creo que eso es todo. Estoy casi seguro de que si no tenemos esperanza, no tiene sentido vivir. Así que, basándome en esta pregunta, creo que siempre tuve la esperanza de que todo saldría bien (Abuela 9).* *La esperanza es creer y transformar. Días mejores (Hermana materna 3).*
**Conductual**: acciones orientadas a la esperanza en los ámbitos físico, psicológico, social y espiritual. Estrategias que pueden promover, fortalecer o debilitar la esperanza familiar.	*Para tener una expectativa de esperanza cuando descubrimos el riesgo, mi suegra y yo fuimos a la capilla, nos arrodillamos y oramos. La estrategia para tener esperanza era creer en Dios* **(Madre 2).** *La estrategia que solía tener esperanza, la adecuada para nosotros, era la unidad, estar juntos, no dejar sola a la persona. A veces me iba de allí, su madre lloraba, le hablaba: ¡Va a salir bien! Creo que la estrategia que usamos fue precisamente esa: unidad, arañarnos unos a otros en la fe, ahí para que todo saliera bien* **(Padre 13).**

A esto le siguió el proceso de codificación de una primera narrativa, que fue realizada por dos investigadores de forma independiente y posteriormente presentada a un tercero. Hicieron falta cuatro rondas de codificación para identificar la congruencia en el proceso. A partir de entonces, los investigadores codificaban las narrativas y las situaciones de divergencias o dudas se resolvían en una reunión. Este proceso aseguraba la fiabilidad de la codificación^16^. Tras el proceso de codificación, fue posible agrupar los códigos para componer las categorías, con el fin de representar y presentar el contenido del análisis deductivo de las narrativas.

Considerando los criterios de cientificidad, aclaramos que, en este estudio, la credibilidad fue posible gracias a análisis rigurosos realizados simultáneamente con el proceso de recogida de datos, respaldados por las narrativas y la construcción de los instrumentos de las relaciones de esperanza. La fiabilidad estaba presente en la descripción detallada del método, siguiendo el *checklist Consolidated Criteria for Reporting Qualitative Research* (COREQ). Se contempló y garantizó la confirmabilidad, considerando la presentación de las fortalezas y limitaciones del estudio^18^ ^-^ ^20^.

### Aspectos éticos

El estudio se llevó a cabo según los aspectos éticos que guían el desarrollo de la investigación con seres humanos en el país, y fue aprobado por el Comité de Ética en la Investigación de la institución proponente bajo el número de opinión 5.119.278 y el Certificado de Presentación de Apreciación Ética (CAAE) número 51866821.4.0000.5545. A todos los participantes se les informó sobre los objetivos de la investigación y completaron el proceso de obtención del consentimiento firmando el Formulario de Consentimiento Informado (FCI), antes de comenzar la recogida de datos. Para garantizar el anonimato, el nombre de cada participante fue reemplazado por la relación con el niño, seguido del orden en que se realizaron las entrevistas, por ejemplo: madre 1, abuelo materno 1, y así sucesivamente.

## Resultados

El análisis de los datos de la investigación nos permitió sintetizar la presentación de los miembros de la familia. De los 28 participantes en el estudio, el 46,4% (13) eran madres biológicas, el 21,4% (6) padres, el 14,3% (4) tías, el 10,7% (3) abuelas, el 3,6% (1) madrinas, el 3,6% (1) hermanas. El rango de edad de los participantes oscilaba entre 25 y 56 años. 

Entre las condiciones de riesgo presentadas por los recién nacidos, identificamos: 15,7% (3) lactantes prematuros, 47,3% (9) prematuridad asociada a gemelos, 10,5% (2) toxoplasmosis congénita y 5,3% (1) para cada uno de los siguientes riesgos: rubéola, derrame pericárdico, sepsis neonatal, prematuridad asociada a sífilis congénita, prematuridad asociada a hidronefrosis neonatal y ascitis. La edad de los niños en el momento de la encuesta oscilaba entre 6 meses y 1 año.

La historia gestacional de las madres estuvo acompañada de riesgo neonatal y, entre los riesgos gestacionales, encontramos: 14,3% (2) de hipertensión gestacional asociada a diabetes gestacional; 14,3% (2) preeclampsia asociada a gemelos y 5,3% (1) para cada uno de los siguientes riesgos: hipertensión gestacional, sífilis gestacional, toxoplasmosis, toxoplasmosis asociada a hipertensión gestacional, hipotiroidismo, preeclampsia asociada a gemelos y desprendimiento placentario, diabetes gestacional asociada a polihidramnios, rubéola, hipertensión gestacional asociada a alteraciones cardíacas y diabetes gestacional asociada a la gemelaridad.

La Figura 2 muestra a los participantes por familia y su relación con el recién nacido, así como los riesgos neonatales y gestacionales que experimenta cada familia.

**Figura 2:** Participantes de la familia, su parentesco con el recién nacido y riesgos gestacionales y neonatales. Divinópolis, MG, Brasil, 2025

Familia	Participantes	Riesgo neonatal	Riesgo gestacional
F1	Padre, Madre	Prematuridad	Síndrome HELLP* y diabetes
F2	Madre, Abuela, Madrina	Sífilis congénita; prematuridad	Sífilis
F3	Madre, Tía	Toxoplasmosis congénita	Toxoplasmosis
F4	Madre, Hermana	Toxoplasmosis congénita	Toxoplasmosis e hipertensión
F5	Madre, Tía	Prematuridad	Preeclampsia y gemelaridad
F6	Padre	Prematuridad, trigemelaridad	Hipertensión, trigemelaridad y diabetes
F7	Madre, Tía	Prematuridad	Hipertensión y diabetes
F8	Madre, Padre	Prematuridad	Hipotiroidismo
F9	Madre, Abuela	Prematuridad; gemelaridad	Preeclampsia, gemelaridad, desprendimiento de placenta, COVID-19
F10	Madre, Padre	Prematuridad	Diabetes, polihidramnios
F11	Madre, Tía	Derrame pericárdico	Hipertensión y alteraciones cardíacas
F12	Madre, Padre	Rubéola congénita	Rubéola
F13	Madre, Padre	Prematuridad	Diabetes gestacional y gemelaridad
F14	Madre, Abuela	Sepsis neonatal	Hipertensión

*HELLP = *Hemolysis, Elevated Liver enzymes, Low Platelets*

Los resultados del análisis deductivo se presentarán de las siguientes categorías: comprensión, juicio e interpretación de la esperanza; Aspectos afectivos y sentimientos en relación con la experiencia vivida; Acciones, comportamientos y estrategias que refuercen la esperanza familiar; Influencia del contexto vital en la experiencia de la (des)esperanza familiar.

### Comprensión, juicio e interpretación sobre la esperanza

En general, los miembros de la familia tienen diferentes interpretaciones de la palabra “Esperanza”. Todos los participantes informaron que la esperanza estuvo presente a lo largo de toda la trayectoria del cuidado del niño, desde el descubrimiento del embarazo de alto riesgo hasta la actualidad. La comprensión de la esperanza se modificó y/o fortaleció en función de la experiencia vivida. Desde el descubrimiento del riesgo gestacional y neonatal, así como tras el nacimiento del recién nacido, se ha evidenciado como un proceso de creencia en un futuro mejor, en el deseo y la expectativa de días mejores.

*Cuando descubrí el riesgo, tenía esperanza de que todo cambiaría. ¿Qué días mejores podrían venir (Madre 10).*

*Cuando nos damos cuenta de que el bebé nació con ese riesgo, entonces entendemos lo que es la esperanza. Siempre pensando en días mejores.*

(Abuela 9)

*Tenía mucha esperanza de que primero el parto fuera bien, ya sabes, con la madre, ya sabía que eran prematuros, que algo podría pasarles. Así que creo que eso es todo, la esperanza de que todo salga bien, que nazcan bien, que nazcan sanos, por mucho que pasemos todo este tiempo en la UCI, siempre tuve la esperanza de que todo saldría bien.*

(Padre 13)

El análisis señala que, con el tiempo, la esperanza se interpreta como una experiencia positiva que puede haberse reforzado por experiencias pasadas, por ejemplo, de descubrir el riesgo gestacional, refiriéndose a la idea de perseverancia y la oportunidad de volver a creer en la vida.

*La esperanza es todo lo que he experimentado. Desde que supe de mi embarazo, podrían llegar prematuramente. Y ahora me doy cuenta de lo importante que era la esperanza para mí.*

(Madre 13)

*Esperanza era lo que creía. Es intentar encontrar la luz realmente desde dentro, creyendo que todo cambiaría. Y, mira, ha cambiado.*

(Hermana paterna 4)

*Es una nueva oportunidad para vivir, cada día. Ellas (las hijas gemelas) me dieron la oportunidad de volver a creer en la vida. Luchar por mi familia.*

(Padre 6)

### Aspectos afectivos y sentimientos en relación con la experiencia vivida

La experiencia familiar en la trayectoria de los riesgos gestacionales y neonatales se construyó a partir de sentimientos y emociones ambiguos y contradictorios, favorecidos o no al mantenimiento de la esperanza y que han cambiado con el tiempo. El análisis nos permite evidenciar que hubo una resignificación de la situación vivida, basada en la temporalidad, y esto hizo que los miembros de la familia mostraran un cambio en sus sentimientos. 

En el contexto del descubrimiento del riesgo que implicaba el embarazo y el recién nacido, los sentimientos se reportaron inicialmente como negativos, con miedo, ansiedad, aprensión, desesperación, tristeza, sorpresa, desconfianza, culpa, ira.

*Cuando supe del riesgo que tenía, me sentí muy molesta y muy aprensiva.*

(Madre 1)

*Creo que la ansiedad se apoderó de mí. Estaba muy ansioso. Esto hizo que mi esperanza se tambaleara.*

(Madre 8)

*No puedo hablar de nada más que de miedo. Incluso cuando nació tenía miedo, todo era extraño.*

(Padre 12)

*Vaya, me sentí muy culpable. Sabiendo que tendría, podría haber, un riesgo por mi culpa.*

(Madre 4)

A pesar de las incertidumbres, tras el nacimiento del recién nacido, se reportaron sentimientos como felicidad, amor, confianza y alivio. Para la mayoría de los miembros de la familia, estos sentimientos permanecen hoy en día.

*Mira, después de que pasó la tormenta, vi que ella había nacido bien, el alivio, la felicidad, me estaban invadiendo.*

(Madre 14)

*Lo que más me domina hasta hoy es la sensación de felicidad, ¿verdad? Cuando vi que estaba bien tras salir de la UCI, se derramaron muchas lágrimas de alegría. Hoy eso es todo, solo alegría.*

(Abuelo materno 14)

Algunos miembros de la familia informan del mantenimiento de sentimientos positivos, como la felicidad y el amor, a lo largo de la experiencia de cuidar al recién nacido, desde el descubrimiento del riesgo gestacional y/o neonatal.

*Cuando lo descubrí, era solo amor. Sentía amor todo el tiempo. Hasta hoy.*

(Madre 10)

*Creo que, a pesar de todo, el sentimiento que más prevalece es la felicidad. Esto me dio esperanza.*

(Padre 10)

### Acciones, comportamientos y estrategias que refuerzan la esperanza familiar

La búsqueda de estrategias y fuentes de esperanza, desde el embarazo y después del nacimiento del recién nacido, implicaba principalmente prácticas de espiritualidad y habilidades para el desarrollo de relaciones intrapersonales. Estas estrategias han estado presentes desde el descubrimiento del riesgo y, con el tiempo, se han reforzado.

Los familiares utilizan estrategias que buscan fortalecer el ámbito espiritual y religioso, a través de la fe en Dios, la oración y las visitas a la iglesia. Estas estrategias han estado presentes desde el descubrimiento de embarazos de alto riesgo y se han reforzado hasta la actualidad.

*Lo que antes tenía esperanza era Jesús. Recé mucho. Era Dios delante.*

(Madre 1)

*Usé a Dios para luchar contra esto, y entonces llegó la esperanza. Y hasta hoy, la fe en Dios prevalece, sin duda.*

(Padre 1)

*Siempre he tenido mucha fe, mucha fe, ¿sabes? Desde que lo descubrí, descubrí el riesgo. Recé mucho, siempre iba a misa, y hoy aún más, estoy agradecida.*

(Madre 4)

*Las estrategias que me ayudaron a tener esperanza fueron fortalecer mi espiritualidad, buscar más la iglesia, esto me ayuda hasta hoy .*

(Madre 11)

Las relaciones intrapersonales de implicación consigo mismo también se consideraban habilidades desarrolladas para generar esperanza. Los miembros de la familia informan haber sido su propio punto de apoyo y acción, especialmente la madre, en la experiencia del embarazo y el nacimiento del recién nacido en riesgo.

*Tenía que ganar, era muy fuerte allí en ese momento para poder ganar.*

(Madre 6)

*Tenemos que llevar el embarazo tan lejos como sea posible y todo irá bien. Ahí fue donde fui como, ya sabes, teniendo más esperanza .*

(Madre 11)

Se identificaron resiliencia y control emocional, así como la autoconfianza para seguir cuidando al recién nacido, con el fin de perseguir el objetivo de mantener la esperanza en la actualidad.

*Es el pensamiento positivo... Sí, intentando controlar bien mis pensamientos, ¿sabes? Esto me ayuda mucho.*

(Madre 4)

*Después de pasar por todo, digo que aprendí a ser resiliente en todo en la vida. Al menos todo esto sirvió como una experiencia de aprendizaje para mi vida personal también .*

(Tía materna 5)

*Después de todo lo que he pasado, después de todo el estrés, llorar tanto, y creo que ahora puedo superar todo. Me hice más fuerte, más resiliente.*

(Padre 10)

### Influencia del contexto y las situaciones de vida en la experiencia de la (des)esperanza familiar

La experiencia de la esperanza estuvo influenciada por el contexto vivido por los miembros de la familia. El análisis nos permitió identificar que la hospitalización del recién nacido, asociada a la pandemia de COVID-19, marcó las experiencias de esperanza. La hospitalización del recién nacido, debido al riesgo, se describió como un periodo que amenazó la esperanza e incluso generó desesperanza. En ese momento, las familias informan que el contacto con el recién nacido se pospuso y que fue difícil regresar a casa sin la presencia de su hijo o hija.

*Ellas (las gemelas) fueron hospitalizadas, ¿verdad?, eso por sí solo ya era malo. Es muy malo que tengamos que irnos de allí y dejarles a ellos. A veces me iba de allí incrédulo...*

(Padre 13)

*La hospitalización amenazó mi esperanza. Verlos (a sus hijos gemelos) hospitalizados sin saber cuándo se irían, ni si se irían... Solo lloré y nada más.*

(Madre 9)

El contexto de la pandemia influyó negativamente en las experiencias familiares, restringiendo y posponiendo el contacto con el recién nacido tras el nacimiento, aumentando la sensación de inseguridad y preocupación.

*Esta pandemia por sí sola ya es mala, ¿verdad? No podemos hacer las cosas bien... No podía ver mucho a mi hijo ni recogerlo, porque todo era muy frágil. Me sentía impotente.*

(Padre 1)

*Llegamos, la preocupación por su caso no fue suficiente, llegó el COVID que nos obligó a quedarnos Un desastre, ¿verdad? El ambiente hospitalario ya no es bueno, con la pandemia entonces...*

(Padre 10)

El análisis de las narrativas mostró que las experiencias durante el embarazo, el parto, después del parto y en los cuidados neonatales, incluido el presente con el niño, contribuyeron a que las familias resignificaran y construyeran su “experiencia de esperanza”, a pesar de haber vivido momentos de desesperanza. Así, los resultados presentan estas experiencias y sus modificaciones a lo largo del tiempo.

La Figura 3 presenta una síntesis de los principales resultados de esta investigación y destaca los elementos que mantuvieron, fortalecieron o amenazaron la esperanza en un camino marcado por cambios y oscilaciones relacionados con la (des)esperanza.

**Figura 3:** (Des)Esperanza familiar durante el embarazo y cuidados neonatales de alto riesgo. Divinópolis, MG, Brasil, 2025

## Discusión

Los resultados mostraron que las experiencias familiares de esperanza se establecen a partir de la creencia en un futuro mejor, emociones ambiguas, comportamientos que buscan fortalecer la esperanza y el contexto de la vida, y estos factores se modifican con el tiempo.

El análisis de las narrativas nos permite afirmar que las historias de las familias presentan algunas similitudes, como el tipo de riesgo gestacional o neonatal y la estructura familiar. La experiencia de esperanza de cada miembro de la familia fue única, influenciada por el entorno en el que se les inserta, reflejando la dimensión contextual proporcionada en el modelo adoptado^6^.

En este estudio, fue evidente que los riesgos gestacionales maternos iban acompañados de riesgos neonatales. Esto reitera la relevancia de identificar el perfil obstétrico y el riesgo gestacional materno de todos los recién nacidos, favoreciendo el pronóstico, reduciendo las probabilidades de morbilidad y mortalidad, hospitalización y deterioro de su salud a lo largo de la vida^21^ ^-^ ^22^.

Los riesgos gestacionales (hipertensión) y neonatales (prematuridad) prevalentes en esta investigación son similares a los de otras realidades nacionales e internacionales^23^ ^-^ ^24^. Esto reafirma la importancia de analizar cuidadosamente los factores que influyen y predisponen al grupo madre-hijo a resultados negativos.

Los informes de los familiares mostraron que la creencia y el deseo de los participantes por días mejores y las expectativas positivas respecto al futuro se reforzaron y mantuvieron con el tiempo, basándose en todo el contexto experimentado. Desde esta perspectiva, podemos inferir que la cognición^6^, expresada en la comprensión y los juicios dirigidos al futuro, favorecía una construcción realista, a pesar de que el futuro madre-hijo está marcado por incertidumbres.

El momento de estabilización del recién nacido, después de que la familia atraviesa momentos como la hospitalización, que amenazaban la esperanza, colabora para intensificar la expectativa de que el riesgo se superará^25^. Así, a pesar de la experiencia del riesgo, se mantuvo la esperanza, fortaleciendo una creencia positiva. Los datos parecen indicar que los momentos de crisis, como el descubrimiento de riesgos o la hospitalización neonatal, funcionan, dependiendo de las interacciones y el apoyo establecido, como dispositivos que refuerzan el fortalecimiento de la esperanza.

Los estudios muestran que experimentar situaciones de crisis adversas afecta a la esperanza, pero proporciona aprendizaje y reflexiones sobre el sentido de la vida y las actitudes orientadas hacia el futuro^26^ ^-^ ^28^. La temporalidad permitió a cada miembro de la familia reconstruir nuevos significados para las experiencias vividas. Desde esta perspectiva, los informes sobre la percepción de la esperanza dieron paso a un sentido positivo y presente que se mantuvo y fortaleció con la experiencia pasada.

La naturaleza cíclica de la experiencia de la esperanza a lo largo del tiempo corrobora otros estudios^26^ ^-^ ^27^, que han demostrado que la esperanza es un recurso dinámico con muchas caras, y que, en momentos de crisis familiar, como el momento del diagnóstico, la esperanza adquiere una forma incierta, caracterizada por preguntas sobre la enfermedad y la dificultad de la familia para aceptar el diagnóstico. Cuando las familias experimentan el impacto positivo del tratamiento y la enfermedad se estabiliza, la esperanza incierta da paso a la esperanza en la atención, manteniendo una perspectiva positiva en relación con el contexto experimentado^26^ ^-^ ^27^.

En momentos marcados por la incertidumbre, los sentimientos inicialmente expresados por los participantes evidenciaron miedo, angustia y tristeza, ejemplificando la dimensión afectiva que impregna la constitución de la (des)esperanza^6^ ^,^ ^29^ ^-^ ^30^.

Situaciones como la hospitalización eran momentos que suponían una amenaza para la esperanza familiar, intensificaban los sentimientos negativos. Los estudios revelan que las familias, además de tener que enfrentarse a un conjunto de sentimientos negativos, deben lidiar con la “etiqueta” de alto riesgo, que apunta claramente a una diferencia en embarazos y partos^31^ ^-^ ^32^.

El nacimiento de un recién nacido en riesgo provoca en la familia una intensa confrontación entre el bebé imaginario y el bebé real. Las expectativas positivas sobre el niño, que se construyen durante el embarazo, se van desmontando gradualmente y acaban volviéndose complejas para la familia, especialmente para las madres, que se enfrentan a un recién nacido con la posibilidad de presentar complicaciones y enfermedades a lo largo de la vida^33^ ^-^ ^34^. Además de este aspecto, existe el contexto de la hospitalización, que va acompañado de sentimientos negativos^35^.

Los resultados revelan que, en el contexto de la hospitalización, la pandemia de COVID-19 tuvo repercusiones en la experiencia vivida. El aislamiento del recién nacido y las restricciones para ingresar en la UCI neonatal hicieron que la familia, especialmente la madre, sintiera la pérdida de su función materna, lo que dificultaba reconocerse a sí misma como madre^36^. Esto ocurre porque, la mayoría de las veces, el equipo se encarga de ello. Esta situación reforzó sentimientos como el miedo y la inseguridad, contribuyendo a amenazar las esperanzas familiares^37^. Esta evidencia nos permite inferir que incluir a la familia en el cuidado, como la del CCF, no es solo una recomendación, ya que mejora el pronóstico neonatal, fortalece la sensación de utilidad y puede contribuir al fortalecimiento de la esperanza.

El momento de la pandemia que acompañó a la hospitalización del recién nacido contribuyó al distanciamiento de la madre de su vida diaria, lo que puede afectar negativamente a su salud mental, generando sentimientos que favorecen la desesperanza^38^ ^-^ ^40^.

También en el contexto de la dimensión afectiva de la esperanza, en momentos de estabilización del riesgo neonatal y en el alta hospitalaria del recién nacido, hay una resignificación de la experiencia vivida. Se ha identificado que emociones positivas como el amor, el alivio y la felicidad se convierten en recursos que impulsan la esperanza^6^ ^,^ ^41^.

 Por otro lado, en algunas situaciones, la condición de riesgo gestacional y neonatal no impidió que el familiar buscara mantener sentimientos positivos durante el proceso. En este estudio, los sentimientos positivos contribuyeron a afrontar situaciones que implicaban riesgo gestacional y neonatal y a superar dificultades^42^ ^-^ ^43^.

Es oportuno para la familia crear estrategias de afrontamiento y, en este estudio, a pesar de la esperanza incierta y la dimensión afectiva en el descubrimiento del riesgo, centrado en los sentimientos negativos, los miembros de la familia reportaron resignar sus emociones manteniendo sus esperanzas a través de la búsqueda de la espiritualidad, una estrategia importante para la elaboración de emociones positivas y la toma de decisiones^6^.

La experiencia familiar en el contexto del riesgo gestacional y neonatal implica sentimientos negativos, que se superan a través de creencias religiosas, que constituyen estrategias que fortalecen la esperanza. Se fortalecen mediante prácticas de oración, asistencia a la iglesia y fe en Dios^44^.

En este contexto, basándose en la dimensión conductual^6^, entre las acciones que contribuyeron al mantenimiento o fortalecimiento de la esperanza de las familias participantes, estuvo la creencia religiosa, una estrategia que se fue fortaleciendo con el tiempo^45^. Al trasladar esta comprensión al contexto del presente estudio, se percibe que los miembros de la familia, al priorizar sus creencias religiosas en sus vidas, pueden buscar una justificación para el contexto en el que se encuentran, lo que facilite afrontar la situación actual.

Los participantes afirmaron que es importante satisfacer sus necesidades espirituales a lo largo del proceso experimentado para fortalecer el cuidado de los recién nacidos. Sin embargo, los estudios indican que las cuestiones sobre creencias religiosas y la valorización de la espiritualidad como recursos para la atención aún no son rutinarias en el enfoque de los profesionales sanitarios^41^ ^,^ ^45^ ^-^ ^46^.

La literatura ha revelado que la mayoría de los profesionales de los equipos sanitarios no son conscientes de la relevancia de las prácticas espirituales para el tratamiento de la salud, o tienen la idea de que tal enfoque no forma parte de su función, y aún existen situaciones en las que el propio profesional de la salud tiene dificultades para discutir el tema^45^ ^,^ ^47^ ^-^ ^48^. Desde esta perspectiva, inferimos que es necesario que los enfermeros reconozcan que la espiritualidad es una dimensión humana, que debería formar parte del cuidado.

En este estudio, identificamos que, en el descubrimiento del riesgo gestacional y neonatal, la esperanza se construye y percibe en el ámbito espiritual de manera optimista, con la perspectiva de un futuro en el que la situación pasa pronto, animando a los familiares a llevar a cabo acciones dirigidas hacia objetivos de esperanza^45^. Estos sentimientos son comprensibles, dado que la esperanza consiste en una experiencia subjetivamente humana, que promueve el bienestar espiritual y sostiene a estas familias motivándolas a desarrollar planes y continuar con el cuidado^26^. Por lo tanto, la esperanza se fortalece en la fe y es esencial para la vida; permite y fortalece a la persona cuando se percibe a sí misma en control de su existencia^49^.

El análisis de las narrativas indica que, entre las estrategias para mantener la esperanza, se encuentra el fortalecimiento de las habilidades intrapersonales de autoconfianza y resiliencia, ejemplificando la dimensión conductual del modelo^6^. Se ha demostrado que la resiliencia es oportuna para afrontar situaciones estresantes dentro del contexto familiar porque contribuye a comportamientos reflexivos y flexibles, que reflejan la experiencia vivida^50^. En la mayoría de los casos, estos comportamientos estaban relacionados con el aprendizaje de lo que se había experimentado en el pasado, desde el descubrimiento del riesgo gestacional.

Un estudio realizado con familias de niños con enfermedades crónicas y profesionales muestra que la comunicación abierta y receptiva entre los miembros de la familia, el apoyo mutuo y una aproximación a la fe religiosa son importantes para que la familia lleve a cabo movimientos espontáneos en el proceso de resiliencia familiar^51^. Los resultados de esta investigación nos permiten afirmar que las relaciones intrapersonales de autoconfianza, habilidades socioemocionales y resiliencia han demostrado ser la base para mantener conductas esperanzadoras.

Este estudio tiene fortalezas y limitaciones. Se consideran puntos fuertes los siguientes: la inclusión de la unidad familiar como componente central de esta investigación y la implicación de otros miembros de la familia del recién nacido, además de la madre; realizando entrevistas en historia oral y la posterior construcción de narrativas, que ayudaron a comprender la historia y el contexto en el que se insertaban las familias y los recién nacidos; El rigor utilizado en el desarrollo de este estudio junto con la descripción detallada del método, que puede ayudar en futuras investigaciones cualitativas y el uso de un marco teórico, que permita un análisis más profundo de los datos.

Como limitación, se reconoce que los resultados tratados aquí se refieren a un grupo de personas expuestas a una red de atención específica y que, en otros lugares, se pueden encontrar otros resultados adicionales. También se entiende que la participación de los profesionales sanitarios que asistieron a estas familias durante la experiencia podría complementar los resultados descritos. Por tanto, futuros estudios que traten estas dimensiones son oportunos. Además, existe la preocupación de llevar a cabo nuevos estudios que busquen identificar y comprender cómo se transmite la enseñanza sobre la esperanza a los estudiantes en el área de la salud, teniendo en cuenta la importancia de este tema para la atención.

Sin embargo, los resultados de este estudio pueden ayudar a los profesionales sanitarios en sus prácticas, en la planificación de la atención familiar sistémica, en el reconocimiento de la esperanza como un recurso familiar esencial y dinámico, en la identificación de las barreras encontradas e implementando estrategias para superarlas. Además, los resultados contribuyen a la comprensión de que la esperanza debe evaluarse en diferentes conjunturas y situaciones, y está relacionada con el contexto, los comportamientos, las relaciones establecidas y los sentimientos a lo largo del tiempo.

## Conclusión

Las evidencias sobre las experiencias familiares de esperanza en el contexto del embarazo y el cuidado de recién nacidos en riesgo contribuyeron a reforzar el carácter dinámico de la esperanza basado en las diferentes situaciones experimentadas. Las experiencias de la (des)desesperanza familiar se caracterizan por percepciones, comportamientos y sentimientos, y están influenciadas por las relaciones establecidas, creencias y emociones, además del contexto en el que se encuentran.

Al principio de la experiencia del riesgo, ya sea gestacional o neonatal, la percepción de esperanza se debilita, evidenciada por la incertidumbre, los sentimientos negativos y los deseos de días mejores. Situaciones como la hospitalización y la pandemia de COVID-19 fueron momentos que permitieron la desesperanza familiar, aumentando los sentimientos negativos en relación con lo que experimentaron. Con el paso del tiempo y la disminución en las situaciones más críticas, el alta hospitalaria del recién nacido y el seguimiento de la atención, la experiencia de la esperanza familiar se modifica y fortalece, mediante el mantenimiento de la comprensión de la esperanza, que se fortalece con las experiencias pasadas. Las estrategias y comportamientos, como la creencia religiosa y el desarrollo de habilidades intrapersonales en las relaciones, se fortalecen y mantienen con el tiempo, contribuyendo al cambio de sentimientos positivos y a la continuidad del cuidado del recién nacido. 

En este sentido, es necesario que los profesionales sean capaces de identificar situaciones de desesperanza familiar en el contexto del riesgo gestacional y llevar a cabo acciones concretas para fortalecer las experiencias de esperanza, tales como: identificar los sentimientos que experimentan las familias y apoyar sentimientos positivos y realistas sobre la situación, mediante escucha atenta y bienvenida; ayudar a fortalecer estrategias y comportamientos de esperanza que buscan fortalecerla, como fortalecer la espiritualidad y la autoconfianza, y a través de actitudes de ánimo familiar, como compartir información y empatía. Hay indicios de que estas estrategias pueden contribuir positivamente a las experiencias familiares y resignificar el contexto vivido.

## Data Availability

Los conjuntos de datos relacionados con este artículo estarán disponibles previa solicitud al autor correspondiente.
